# TEFM drives cartilage degeneration in osteoarthritis via the ROS-AKT/NF-κB signaling axis

**DOI:** 10.3389/fimmu.2026.1961836

**Published:** 2026-10-07

**Authors:** Kanghua Yang, Meifeng Lu, Runting Xiao, Shuai Xu, Yulong Ouyang, Guicai Sun

**Affiliations:** 1 Department of Sports Medicine, Orthopaedic Hospital, The First Affiliated Hospital, Jiangxi Medical College, Nanchang University, Nanchang, Jiangxi, China; 2 Ganzhou Hospital-Nanfang Hospital, Southern Medical University (Ganzhou People’s Hospital), Ganzhou, Jiangxi, China

**Keywords:** chondrocyte, NF-κB, osteoarthritis, reactive oxygen species, TEFM

## Abstract

**Objective:**

To explore the role and underlying mechanism of mitochondrial transcription elongation factor TEFM in osteoarthritis (OA) cartilage degeneration.

**Methods:**

Bioinformatics analysis based on the GSE114007 human knee cartilage dataset and two-sample Mendelian randomization (MR) analysis were performed to evaluate the association between TEFM and knee OA. Human cartilage specimens were collected to verify TEFM expression patterns. Lentivirus-mediated TEFM knockdown was performed in ATDC5 chondrocytes, and an *in vitro* inflammatory injury model was established by interleukin-1β (IL-1β) stimulation. The effects of TEFM on chondrocyte function were determined by RT-qPCR, Western blotting, toluidine blue staining, and immunofluorescence staining. Transcriptome sequencing (RNA-seq), reactive oxygen species (ROS) scavenging, specific pathway inhibition, and H_2_O_2_ rescue assays were employed to elucidate TEFM-mediated signaling cascades. For *in vivo* validation, a destabilization of the medial meniscus (DMM)-induced OA mouse model was established, and adeno-associated virus serotype 9 carrying sh-TEFM (AAV9-sh-TEFM) was administered via intra-articular injection. Pathological changes in cartilage and subchondral bone were assessed by micro-CT and histological staining.

**Results:**

TEFM expression was significantly upregulated in OA cartilage, and MR analysis supported a potential causal association with knee OA risk. *In vitro* experiments demonstrated that TEFM knockdown ameliorated chondrocyte dysfunction, attenuated mitochondrial oxidative stress, and suppressed AKT/NF-κB pathway activation. Mechanistically, NAC treatment and H_2_O_2_ rescue assays verified that ROS serve as a critical upstream mediator linking TEFM to AKT/NF-κB signaling, and specific inhibitor assays further confirmed the functional necessity of the AKT/NF-κB cascade in TEFM-mediated chondrocyte injury. *In vivo* studies revealed that intra-articular TEFM silencing alleviated cartilage degeneration and subchondral bone pathological damage in OA mice, accompanied by inhibition of the AKT/NF-κB pathway.

**Conclusion:**

TEFM facilitates OA cartilage degeneration via the ROS-AKT/NF-κB axis, which provides a novel therapeutic target for OA.

## 1 Introduction

Osteoarthritis (OA) is a prevalent chronic joint disorder primarily characterized by articular cartilage degeneration, subchondral bone sclerosis and synovial inflammation, and represents one of the leading causes of joint pain and physical disability in middle-aged and elderly populations worldwide (1, 2). The 2021 Global Burden of Disease (GBD) Study reported that the global number of patients with OA reached 595 million in 2020, and its prevalence continues to rise driven by accelerating population aging (1). The pathogenesis of OA is multifactorial, involving aberrant mechanical loading, chronic inflammatory responses, chondrocyte dysfunction and extracellular matrix degradation (3, 4). Accumulating evidence has highlighted the central roles of disrupted chondrocyte metabolism and mitochondrial dysfunction in OA initiation and progression (5–7). As the primary resident cell type in articular cartilage, chondrocytes maintain the balance between matrix anabolism and catabolism through tightly regulated energy metabolism (7). Mitochondria serve as the central hub of cellular energy supply and redox homeostasis; their dysfunction leads to excessive production and accumulation of reactive oxygen species (ROS), which further activates downstream inflammatory signaling cascades and ultimately drives cartilage degeneration and OA progression.

Mitochondria possess their own semi-autonomous circular genome, and their transcription relies on the coordinated action of multiple nuclear-encoded factors (8). Mitochondrial transcription elongation factor (TEFM), a pivotal mitochondrial transcriptional regulator localized within the mitochondrial matrix, interacts with mitochondrial RNA polymerase (POLRMT) to facilitate the elongation of mitochondrial DNA (mtDNA) transcripts, thereby ensuring normal expression of mitochondrially encoded subunits of oxidative phosphorylation (OXPHOS) complexes (8–10). Substantial evidence has established the vital roles of TEFM in sustaining mitochondrial respiratory chain function and in governing cellular energy metabolism and redox homeostasis (9, 11, 12). TEFM variants are documented to cause respiratory chain deficiency and childhood-onset mitochondrial neurological disorders (11). Moreover, TEFM upregulation has been documented in various human malignancies, including lung adenocarcinoma, hepatocellular carcinoma, endometrial carcinoma and glioma, and correlates with tumor progression and poor clinical prognosis (13–15). However, to date, no study has systematically characterized the expression profile and pathological relevance of TEFM in OA cartilage and chondrocytes.

Mitochondrial dysfunction is a key driver of excessive ROS generation during OA pathogenesis (16). Excess ROS activates IκB kinase (IKK) and drives the phosphorylation and sequential degradation of inhibitor of nuclear factor-κB (IκB), thereby promoting nuclear translocation of the NF-κB p65 subunit. This in turn triggers transcription of the genes encoding downstream pro-inflammatory cytokines (IL-1β, IL-6, TNF-α, etc.) and matrix metalloproteinases (MMPs), which ultimately exacerbates extracellular matrix breakdown and articular cartilage destruction (16–18). Previous oncological studies have demonstrated that TEFM upregulation impairs mitochondrial function, enhances ROS production and reduces mitochondrial membrane potential (15). However, chondrocytes chronically reside in the hypoxic joint microenvironment, and their mitochondrial energy metabolism and oxidative stress tolerance differ markedly from those in epithelial tumor cells. As such, experimental evidence defining TEFM’s functional roles and regulatory mechanisms in cartilage degeneration remains limited.

To date, few studies have systematically delineated the expression profiles, biological functions and downstream molecular mechanisms of TEFM in OA cartilage and chondrocytes. This study adopts a multi-tiered validation strategy encompassing bioinformatic analysis, mendelian randomization (MR), human articular cartilage specimens, *in vitro* chondrocyte functional assays, and *in vivo* destabilization of the medial meniscus (DMM)-induced OA mouse model. We aim to elucidate the pathological role of TEFM in OA and delineate the full TEFM-ROS-AKT/NF-κB regulatory axis. This work advances the understanding of OA pathogenesis from the novel perspective of mitochondrial transcriptional regulation, and provides a theoretical basis for developing novel targeted therapeutic strategies for OA.

## 2 Materials and methods

### 2.1 Bioinformatic analysis

The human knee osteoarthritis transcriptome dataset GSE114007 was retrieved from the Gene Expression Omnibus (GEO) public repository (http://www.ncbi.nlm.nih.gov/geo/), which contained 18 normal articular cartilage samples and 20 OA lesional cartilage samples. Differential expression gene analysis was performed using the limma package in R software (v4.5.2), with screening thresholds set as |log_2_fold change (FC)| ≥ 0.585 (FC ≥ 1.5) and *P* < 0.05. All samples were stratified into high and low TEFM expression groups according to the median TEFM expression level across the entire cohort. The clusterProfiler package was employed for Gene Ontology (GO) functional enrichment and Kyoto Encyclopedia of Genes and Genomes (KEGG) pathway enrichment analyses. Gene Set Enrichment Analysis (GSEA) was performed against Molecular Signatures Database (MSigDB) gene sets to interrogate enrichment signatures of pathways associated with inflammatory response, mitochondrial function and cartilage matrix metabolism.

Two-sample MR analysis was conducted to infer the causal relationship between TEFM expression and OA susceptibility. The exposure was TEFM gene expression, and summary-level whole-blood eQTL data were extracted from the IEU OpenGWAS database (ID: eqtl-a-ENSG00000172171). The outcome was knee OA risk, with corresponding GWAS summary statistics obtained from IEU OpenGWAS (ID: ebi-a-GCST007090, phenotype: clinically diagnosed knee osteoarthritis). Independent single-nucleotide polymorphisms (SNPs) satisfying *P* < 5×10^-8^, F-statistic > 10 and linkage disequilibrium *r²* < 0.001 were selected as valid instrumental variables. Instrument strength was evaluated using F-statistics approximated as (β/SE)², and the variance explained by each SNP was estimated as R² = 2 × EAF × (1 − EAF) × β². The multiplicative random-effects inverse variance weighted (IVW) method served as the primary analytical approach to estimate causal effects. Multiple sensitivity analyses including MR-Egger regression, weighted median method, Cochran’s Q heterogeneity test, and leave-one-out analysis, were performed to assess the robustness of the identified causal associations (19).

### 2.2 Collection and processing of clinical cartilage specimens

A total of 12 human cartilage specimens were collected in this study: 6 OA lesional cartilage samples were obtained from patients with severe knee OA undergoing total knee arthroplasty (TKA), and 6 normal cartilage samples were collected from patients undergoing amputation due to trauma. The two groups were well matched in baseline characteristics including age and comorbidities. Cartilage in the control group was radiographically confirmed as Kellgren-Lawrence (KL) grade 0. Detailed sample information is summarized in **Supplementary Table S1**. All participants signed written informed consent, and the study protocol was reviewed and approved by the Institutional Ethics Committee of our hospital (Ethics Approval No. (2025)CDYFYYLK(12-008)). After isolation, all specimens were immediately fixed in 4% paraformaldehyde, followed by routine paraffin embedding and sectioning for subsequent hematoxylin-eosin (H&E) staining, Safranin O-Fast Green (SO&FG) staining and immunohistochemistry (IHC). A portion of fresh cartilage specimens were snap-frozen in liquid nitrogen immediately after harvest and stored at −80 °C for total RNA and total protein extraction. For molecular assays, 3 ankle cartilage specimens from the control group and 3 knee cartilage specimens from the OA group were selected for total RNA and protein extraction.

### 2.3 Cell culture and treatment

The mouse chondrocyte cell line ATDC5 (Zishan Biotechnology, STCC20002P) was used for *in vitro* experiments. Cells were cultured in DMEM/F12 medium supplemented with 10% fetal bovine serum (Servicebio, G8003) and 1% penicillin-streptomycin double antibiotics (Servicebio, G4003) at 37 °C in a humidified incubator with 5% CO_2_. To establish an *in vitro* OA inflammatory injury model, cells were stimulated with 10 ng/mL recombinant mouse IL-1β (PeproTech, 211-11B-10UG) for 24 h. Lentiviral vectors encoding short hairpin RNA targeting the TEFM gene (sh-TEFM) and negative control lentivirus (sh-NC) were constructed and packaged by Shanghai Hanheng Biotechnology Co., Ltd. ATDC5 cells were infected at a multiplicity of infection (MOI) of 50, followed by screening with 2 μg/mL puromycin (Servicebio, G4017-1ML) for 7–10 days to generate stably TEFM-knockdown cell lines. For rescue and pathway validation assays, cells were pretreated with 5 mM N-acetylcysteine (NAC, MCE, HY-B0215) 1 h or 10 µM MK-2206 (selective AKT inhibitor, Glpbio, GC16304) for 2 h, followed by 10 ng/mL IL-1β stimulation for another 24 h. For rescue experiments, stable TEFM-knockdown cells were co-treated with 100 nM phorbol 12-myristate 13-acetate (PMA, Servicebio, G2272-100UL) or 200 µM exogenous hydrogen peroxide (H_2_O_2_) together with IL-1β for 24 h, respectively.

### 2.4 Quantitative real-time PCR

Total RNA was extracted from cells or tissues using a spin-column RNA extraction kit (Beyotime, R0026) according to the manufacturer’s instructions. RNA concentration and purity were determined on a NanoDrop spectrophotometer, and samples with an A260/A280 ratio within 1.8–2.0 were retained for subsequent experiments. Genomic DNA elimination were performed first, followed by reverse transcription using the PerfectStart^®^ Uni RT&qPCR Kit (TransGen Biotech, AUQ-01) to synthesize first-strand cDNA. Quantitative PCR amplification was carried out on a real-time fluorescence quantitative PCR system (Bio-Rad, USA). All primer sequences were synthesized by Servicebio (**Table 1**). Glyceraldehyde-3-phosphate dehydrogenase (GAPDH) was used as the internal reference gene, and the 2^-^ΔΔCt method was applied to calculate the relative expression levels of target genes including TEFM, COL2A1 and MMP13. All assays were performed in triplicate with three independent biological replicates.

**Table 1:** Primer sequence.

Gene	Forward primer	Reverse primer
COL2a1	GGAAGAGCGGAGACTACTGGATT	GCTCTTGCTGCTCCACCAGTT
TEFM	CATCAAGCCAGGTGTAGAGCAA	ACAGTCGGTCTCCGGTCAGG
GAPDH	CCTCGTCCCGTAGACAAAATG	TGAGGTCAATGAAGGGGTCGT
MMP13	GTTTTCTTTATGGTCCAGGCGAT	GACTGGTAATGGCATCAAGGGA

### 2.5 Western blot

Total protein was extracted from cells or tissues using RIPA lysis buffer (Solarbio, R0020) supplemented with 1% protease inhibitor cocktail (Servicebio, G2008) and phosphatase inhibitor cocktail (Servicebio, G2007). Protein concentration was quantified with a BCA protein assay kit (Servicebio, G2026). Equal amounts of protein were separated by sodium dodecyl sulfate-polyacrylamide gel electrophoresis (SDS-PAGE) and transferred onto polyvinylidene difluoride (PVDF) membranes (Seven Biotech, SW221-02). The membranes were blocked with protein-free rapid blocking buffer (Servicebio, G2052-500ML) at room temperature for 30 min. Subsequently, membranes were incubated overnight at 4 °C with primary antibodies at the indicated dilutions: TEFM (Epizyme, P107794, 1:1000), type II collagen (COL II, Nature Biosciences, A39981, 1:1000), matrix metalloproteinase 13 (MMP13, Nature Biosciences, A35406, 1:1000), phosphorylated AKT at Ser473 (p-AKT, Proteintech, 80455-1-RR, 1:1000), total AKT (Proteintech, 10176-2-AP, 1:1000), phosphorylated NF-κB p65 at Ser468 (p-p65, Proteintech, 82335-1-RR, 1:1000), total NF-κB p65 (Proteintech, 80979-1-RR, 1:1000), and GAPDH (Proteintech, 60004-1-Ig, 1:50000). Following overnight incubation, membranes were incubated with corresponding horseradish peroxidase (HRP)-conjugated secondary antibodies (1:5000; rabbit secondary antibody: SA00001-2, mouse secondary antibody: SA00001-1, Proteintech) at room temperature for 2 h. Protein bands were visualized using an enhanced chemiluminescence (ECL) kit (Abbkine, BMU102), and band grayscale intensities were quantified using ImageJ software (version 1.54, USA). Notably, for MMP13 protein quantification, the 54 kDa band corresponding to the inactive zymogen form (pro-MMP13) was selected for analysis, as this band exhibited stable expression and consistent band intensity across all samples and biological replicates. The 45 kDa band corresponding to the proteolytically cleaved active form of MMP13 was excluded from quantitative analysis due to its faint signal intensity and inconsistent detectability among samples. This quantification strategy is consistent with standard practice in osteoarthritis research.

### 2.6 IHC staining

Paraffin sections were deparaffinized and rehydrated, followed by heat-mediated antigen retrieval in citrate buffer (pH 6.0). Endogenous peroxidase activity was blocked by incubation with 3% H_2_O_2_ for 15 min at room temperature, and non-specific binding was blocked with 5% BSA for 30 min at room temperature. Sections were incubated overnight at 4 °C in a humidified chamber with primary antibodies at a dilution of 1:100, including COL II (Nature Biosciences, A39981), TEFM (Epizyme, P107794), MMP13 (Nature Biosciences, A35406), p-AKT (Proteintech, 80455-1-RR), and p-p65 (Proteintech, 82335-1-RR). Following overnight incubation, sections were incubated with the corresponding horseradish peroxidase (HRP)-conjugated secondary antibodies, and signals were developed using 3,3’-diaminobenzidine (DAB) chromogen. Sections were counterstained with hematoxylin, dehydrated, cleared, and mounted with neutral balsam. Images were captured under a light microscope, and the mean optical density (MOD) was quantified using ImageJ software.

### 2.7 IF staining

Cells were seeded into 96-well plates for COL II and MMP13 detection, or glass-bottom confocal dishes for evaluation of p65 nuclear translocation. After adherence, cells were treated according to experimental groups, fixed with 4% paraformaldehyde for 15 min, permeabilized with 0.3% Triton X-100 for 15 min, and blocked with 5% BSA for 1 h at room temperature. Subsequently, cells were incubated overnight at 4 °C with primary antibodies diluted at 1:200: COL II (Nature Biosciences, A39981), MMP13 (Nature Biosciences, A35406), and p65 (Proteintech, 80979-1-RR). Following overnight incubation, corresponding Alexa Fluor-conjugated secondary antibodies (1:200) were added and incubated for 1 h at room temperature in the dark, followed by nuclear counterstaining with DAPI for 5 min. After mounting with anti-fade mounting medium, images of COL II and MMP13 were captured under an inverted fluorescence microscope, while p65 nuclear translocation was visualized and imaged via laser scanning confocal microscopy. Fluorescence intensity was quantified using ImageJ software.

### 2.8 Toluidine blue staining

Chondrocytes were seeded in 96-well plates at a density of 2×10³ cells per well. After drug intervention, the cells were fixed with 4% PFA at room temperature for 15 min. Then, the cells were stained with toluidine blue staining solution (Servicebio, G1032) at room temperature for 5 min, followed by thorough rinsing with PBS to remove background dye. Finally, the cells were observed and images were captured under an optical microscope.

### 2.9 Detection of intracellular ROS

After cells were seeded in culture plates and subjected to IL-1β stimulation and corresponding treatments, intracellular total ROS levels were detected using the 2’,7’-dichlorodihydrofluorescein diacetate (DCFH-DA) fluorescent probe (Servicebio, G1706-100T). The DCFH-DA stock solution was diluted at a ratio of 1:1000 with serum-free medium to prepare the working solution. The cell culture medium was replaced with the probe working solution, and cells were incubated at 37 °C in a cell incubator for 30 min in the dark. Cells were thoroughly washed three times with PBS to remove uninternalized free probes. Subsequently, Hoechst 33342 nuclear staining working solution (Servicebio, G1127-1ML) was added at a final concentration of 1 μg/mL for nuclear counterstaining, followed by incubation at room temperature for 10 min in the dark. Excess dye was washed off with PBS. All procedures were performed under light-protected conditions. Fluorescent images were captured with an inverted fluorescence microscope, and the fluorescence signal intensity was quantitatively analyzed using ImageJ software.

### 2.10 Detection of mitochondrial ROS

Cells were seeded in glass-bottom confocal dishes and received IL-1β stimulation and corresponding lentiviral interventions as described above. Mitochondrial superoxide levels were measured using the MitoSOX™ Red mitochondrial superoxide indicator (Beyotime, S0061S). The MitoSOX Red stock solution was diluted to a final working concentration of 5 μM with pre-warmed serum-free culture medium. After removing the original culture medium, cells were incubated with the probe working solution at 37 °C in a humidified 5% CO_2_ incubator for 30 min in the dark. Cells were then washed three times with pre-warmed PBS to remove excess unbound probe. Subsequently, Hoechst 33342 working solution (Servicebio, G1127-1ML) was added at a final concentration of 1 μg/mL for nuclear counterstaining, followed by incubation at room temperature for 10 min in the dark. Excess dye was removed by washing with PBS, and all procedures were performed under light-protected conditions. Fluorescence images were acquired using a laser scanning confocal microscope, and the mean fluorescence intensity of MitoSOX Red was quantified using ImageJ software.

### 2.11 Detection of mitochondrial membrane potential

Mitochondrial membrane potential was measured using the tetramethylrhodamine methyl ester (TMRM) probe (#C2001S, Beyotime). After cells were treated according to different groups, TMRM stock solution was diluted at a ratio of 1:1000 and added to cells, followed by incubation at 37 °C for 30 min in the dark. After washing with PBS, red fluorescence signals were observed under an inverted fluorescence microscope.

### 2.12 Transmission electron microscopy

Following the indicated treatments, cells were washed three times with pre-chilled PBS, and then fixed with electron microscopy fixative (Servicebio, G1102, China) at 4 °C for 4 h. After three rinses with 0.1 M phosphate buffer (15 min each), samples were post-fixed with 1% (w/v) osmium tetroxide at 4 °C for 1 h. Cells were then dehydrated in an ascending gradient of ethanol (30%, 50%, 70%, 80%, 90%, 95%, and 100%) for 15 min per concentration, followed by gradual infiltration and embedding in epoxy resin. Ultrathin sections with a thickness of 60–80 nm were prepared using an ultramicrotome, collected on copper grids, and subjected to double staining with uranyl acetate and lead citrate for 10 min each. The ultrastructural morphology of mitochondria (including cristae integrity, mitochondrial membrane integrity and overall morphology) was observed and imaged under a transmission electron microscope (Hitachi, HT7800).

### 2.13 Transcriptome sequencing

ATDC5 chondrocytes from three groups (sh-NC, IL-1β + sh-NC, IL-1β + sh-TEFM) were harvested, and total cellular RNA was extracted using the Trizol method. RNA integrity was verified with an Agilent 2100 Bioanalyzer, and samples with an RNA integrity number (RIN) > 7.0 were retained for subsequent cDNA library construction. Library preparation and high-throughput sequencing were performed by Panoson Biotechnology Co., Ltd., and paired-end sequencing (PE150) was conducted on the Illumina NovaSeq 6000 platform. After quality control and filtering of raw sequencing reads, HISAT2 (v2.1.0) was used to map clean reads to the mouse reference genome (mm10), followed by gene expression quantification with StringTie (v2.1.4). Differential expression analysis was implemented via DESeq2, with screening thresholds set as |log_2_FC| > 1 and *P* < 0.05. The clusterProfiler package was adopted for KEGG pathway and GO functional enrichment analyses to screen differentially expressed genes related to inflammation and cartilage metabolism that were reversed upon TEFM knockdown. In addition, GSEA was performed based on global gene expression profiles, focusing on inflammation-associated pathways. Pathways with a normalized enrichment score (NES) and P < 0.05 were defined as significantly enriched.

### 2.14 Animal experiments and *in vivo* intervention

Eight-week-old male C57BL/6J mice weighing 20–22 g were housed in an SPF-grade animal facility and acclimatized for one week prior to experiments. All animal procedures were approved by the Institutional Animal Care and Use Committee of our institute (Ethics Approval No.: CDYFY-IACUC-202512GR078). Mice were randomly assigned to three groups with seven animals per group: Sham group (intra-articular injection of 10 μL normal saline), DMM + AAV-sh-NC group, and DMM + AAV-sh-TEFM group. The DMM surgical OA model was established following standard protocols. One week after surgery, DMM-operated mice received intra-articular injection of 10 μL AAV9 serotype adeno-associated viral suspension carrying AAV-sh-NC or AAV-sh-TEFM (titer = 1 × 10¹² vg/mL, Hanheng Biotechnology) per knee joint. Mice in the Sham group were injected with an equal volume of normal saline at the same time point. All mice were sacrificed at week 9 postoperatively, and operated knee joints were harvested and fixed in 4% paraformaldehyde for subsequent Micro-CT and histological analyses.

### 2.15 Micro-CT analysis of bone microarchitecture

Fixed knee joint specimens were subjected to tomographic scanning using a Micro-CT system (MILabs B.V., the Netherlands). Raw scanning images were preprocessed and three-dimensionally reconstructed with the matching MILabs CT Analyzer software to visualize the spatial distribution and morphological characteristics of trabecular bone. The subchondral bone region of the tibial plateau was selected as the region of interest (ROI). Bone microstructural parameters including bone volume fraction (BV/TV), trabecular number (Tb.N), trabecular thickness (Tb.Th), and trabecular separation (Tb.Sp) were quantified.

### 2.16 Histological evaluation

Mouse knee specimens were fixed in 4% paraformaldehyde for 48 h at room temperature, followed by decalcification in 10% EDTA solution (pH 7.4) for 4 weeks. After gradient dehydration, clearing and paraffin infiltration, tissues were embedded to prepare 4 μm continuous sections. H&E staining, SO&FG staining and IHC staining targeting COL II, MMP13, p-AKT and p-p65 were performed separately. Semi-quantitative double-blind scoring of articular cartilage degeneration was conducted in accordance with the Osteoarthritis Research Society International (OARSI) grading criteria.

### 2.17 Detection of cell proliferation and viability

Cell proliferation activity of ATDC5 cells in each group was assessed using an EdU Cell Proliferation Kit (Beyotime, C0075S). All operations were performed following the manufacturer’s instructions. Briefly, cells were incubated with 10 μM EdU working solution for 2 h, then sequentially subjected to fixation, permeabilization, Click-iT reaction in the dark and DAPI counterstaining. After PBS washing, images were captured under an inverted fluorescence microscope, and the proportion of EdU-positive cells was quantified with ImageJ software.

### 2.18 Statistical analysis

All statistical analyses were performed using GraphPad Prism 9.0 software (GraphPad Software, USA). Measurement data are presented as mean ± standard deviation (SD). Normality was assessed using the Shapiro–Wilk test and homogeneity of variance by the F-test prior to parametric analysis. For comparisons between two groups, independent-samples t-test was used. For quantitative analysis of ROS fluorescence and semi-quantitative analysis of immunofluorescence staining, at least 5 non-overlapping high-power fields were randomly selected per sample for image acquisition. All image acquisition and quantitative analyses were conducted in a blinded fashion; the evaluator was masked to sample grouping and treatment conditions to minimize measurement bias. Statistical significance was set at *P* < 0.05 (*P < 0.05, **P < 0.01, ***P < 0.001, ****P < 0.0001). All cellular and molecular biology experiments were independently repeated with at least three biological replicates to ensure data reliability.

## 3 Results

### 3.1 Bioinformatic analysis and MR validation: TEFM is significantly upregulated in OA and exhibits a potential causal association with elevated disease risk

To identify key regulatory molecules driving OA initiation and progression, differential expression analysis was first performed on the GSE114007 OA-related transcriptomic dataset retrieved from the GEO database. Heatmaps and volcano plots identified abundant differentially expressed genes between normal and OA cartilage, among which TEFM was markedly upregulated in lesional OA cartilage (**Figures 1A–C**). To dissect the functional implications of elevated TEFM in OA, samples were stratified into high- and low-TEFM expression groups based on the median TEFM value for enrichment analysis. Results demonstrated that pathways regulating autophagy, FoxO signaling, mitogen-activated protein kinase (MAPK) signaling, tumor necrosis factor (TNF) signaling, and mitophagy were significantly downregulated in the high-TEFM subgroup. Conversely, pathways associated with cell cycle and ECM-receptor interaction were significantly upregulated in the high-TEFM subgroup (**Figures 1D, E**).

**Figure 1:**
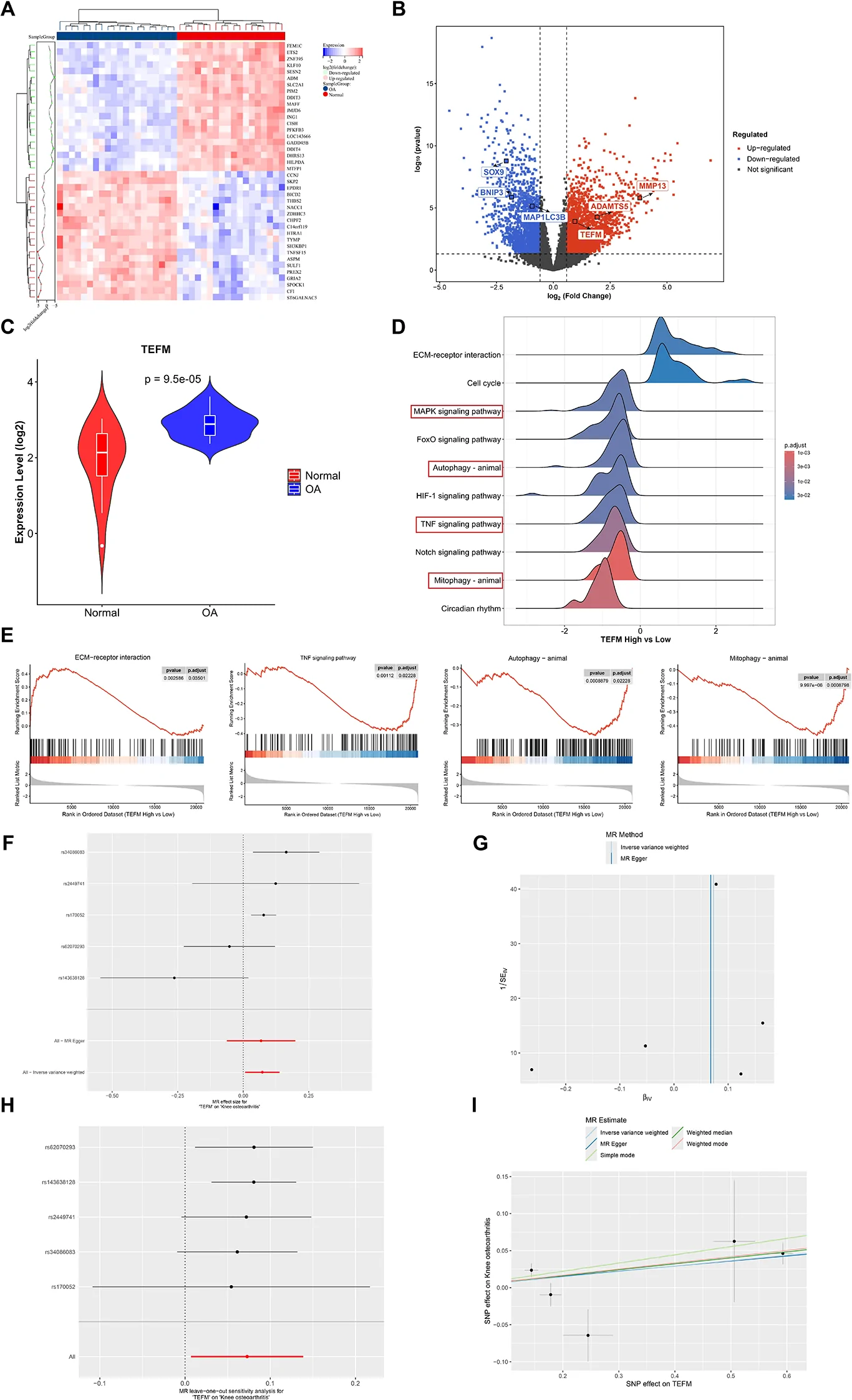
Bioinformatic analysis identifies TEFM as a key upregulated gene in OA cartilage and supports its causal association with knee OA. **(A)** Heatmap of the top differentially expressed genes between normal (n = 18) and OA (n = 20) cartilage in GSE114007 (row-scaled Z-scores). **(B)** Volcano plot of all genes; upregulated (red), downregulated (blue), and non-significant (grey) genes are defined by |log_2_ fold change| ≥ 0.585 and *P* < 0.05. **(C)** Violin plot of TEFM expression between groups (Wilcoxon rank-sum test, *P* = 9.5 × 10^-5^). **(D)** KEGG enrichment of genes stratified by median TEFM expression; ridge plots are colored by adjusted P value. The x-axis shows the log_2_ fold change (TEFM high vs. low): right-shifted ridges indicate pathway activation in the high-TEFM subgroup, and left-shifted ridges indicate suppression. **(E)** GSEA of TEFM-associated pathways. Genes are ranked by the log_2_ fold change (TEFM high vs. low); a positive NES indicates enrichment in the high-TEFM subgroup, and a negative NES indicates enrichment in the low-TEFM subgroup. **(F–I)** Two-sample MR of TEFM expression (eQTLs, eqtl-a-ENSG00000172171) on knee OA (ebi-a-GCST007090) using five instrumental SNPs: **(F)** forest plot; **(G)** funnel plot; **(H)** leave-one-out analysis; **(I)** scatter plot of SNP effect sizes with regression slopes for five MR methods. Error bars denote 95% confidence intervals.

Two-sample MR analysis was subsequently conducted to verify the causal relationship between TEFM and OA. A total of five SNPs located within the cis-region of the TEFM gene (chromosome 17) were selected as instrumental variables. All SNPs met the genome-wide significance threshold (P < 5 × 10^-8^) and were in linkage equilibrium. The strength of the instruments was further assessed: the F statistic of every individual SNP exceeded 10 (ranging from 30.1 to 1035.3; mean F = 296.7), and the overall mean F statistic was well above the conventional weak-instrument threshold (F = 10), indicating strong instruments with a low risk of weak instrument bias. Collectively, the five SNPs jointly explained approximately 10% of the variance in TEFM expression, a level considered reasonable for cis-eQTL-based instruments. As Cochran’s Q test indicated marginal heterogeneity across instruments (Q = 9.566, df = 4, *P* = 0.048), multiplicative random-effects IVW was adopted as the primary estimator, which revealed that genetically predicted TEFM expression was significantly positively associated with knee OA risk (OR = 1.076, 95% CI: 1.007–1.149, *P* = 0.030), implying that elevated TEFM constitutes a potential pathogenic factor for knee OA. Sensitivity analyses supported the robustness of this causal inference: the intercept term of MR-Egger regression showed no statistically significant deviation from zero (*P* = 0.933), indicating no detectable horizontal pleiotropy; the weighted median approach yielded effect estimates consistent with the IVW results (OR = 1.084, 95% CI: 1.036–1.134, *P* = 4.7 × 10^-4^), as did the weighted mode (OR = 1.087, 95% CI: 1.036–1.142, *P* = 0.028). Leave-one-out analysis confirmed that no single SNP exerted an outsized influence on the overall causal effect. Collectively, the MR analyses support a potential causal association between TEFM expression and knee OA susceptibility (**Figures 1F–I**), suggesting that abnormally increased TEFM may facilitate OA progression by modulating mitochondrial homeostasis and inflammatory responses.

### 3.2 TEFM is upregulated in human OA cartilage tissues

To validate the bioinformatic findings, six human normal cartilage specimens and six OA lesional cartilage specimens were collected for histological staining and molecular biological validation. H&E staining revealed typical OA morphological alterations, including surface fibrillation, cartilage fissures, chondrocyte clustering and disorganized zonal architecture, while SO&FG staining demonstrated marked proteoglycan loss in OA cartilage (**Figure 2A**). IHC showed that the COL II–positive area was significantly reduced, whereas the TEFM-positive area was markedly expanded in OA cartilage relative to controls (**Figures 2A–C**). Consistently, qRT-PCR further verified that compared with normal controls, mRNA levels of COL II were drastically decreased, while those of MMP13 and TEFM were notably elevated in OA cartilage tissues (**Figures 2D–F**). WB analysis showed consistent protein expression trends: COL II protein was downregulated, and MMP13 as well as TEFM proteins were upregulated in OA tissues (**Figures 2G–J**). Collectively, these data indicate that TEFM is highly expressed in human OA cartilage, and its upregulation is synchronized with the expression changes of classic cartilage degeneration markers (decreased COL II and increased MMP13), suggesting a potential involvement of TEFM in OA cartilage degeneration.

**Figure 2:**
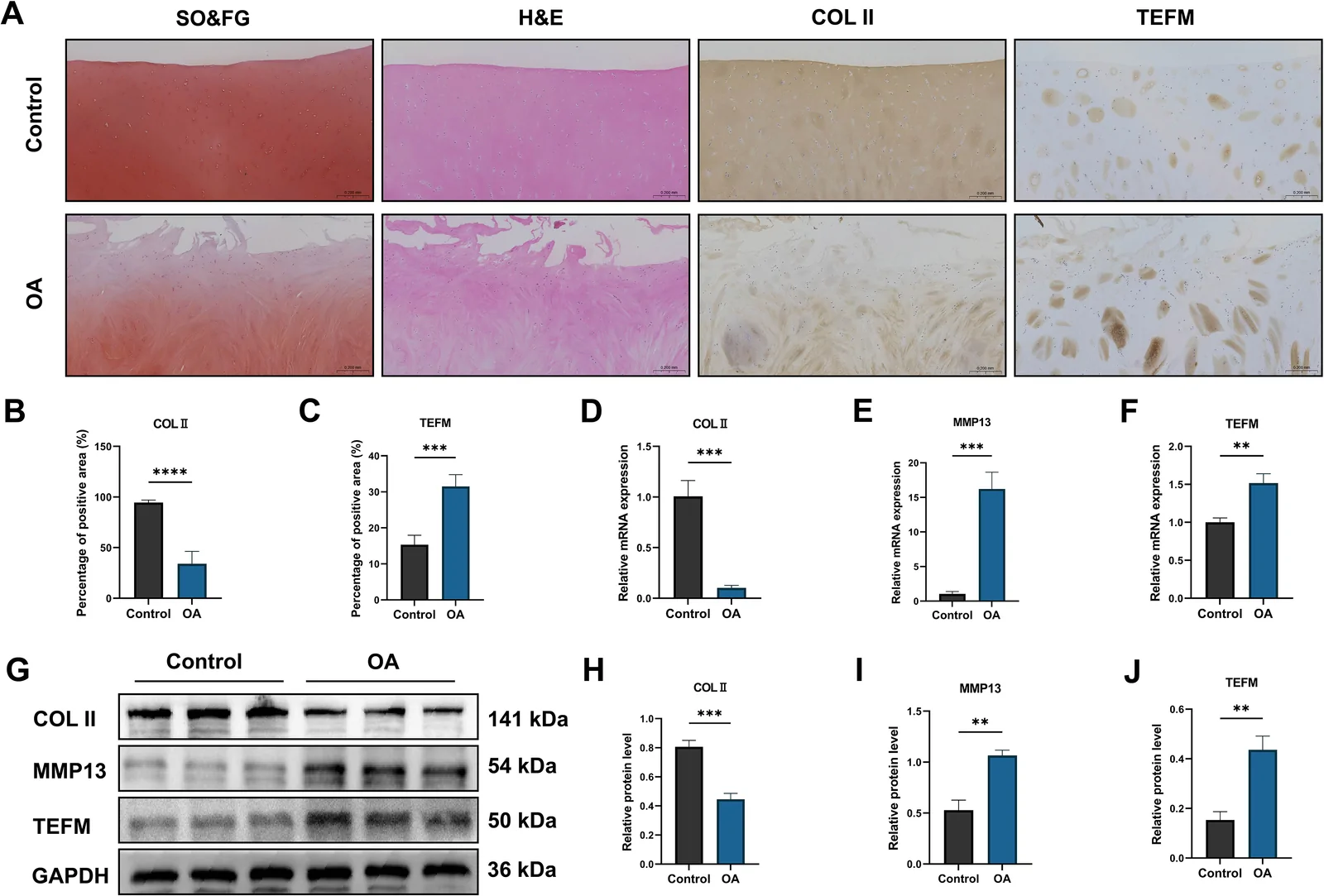
TEFM is upregulated in human osteoarthritic cartilage tissues. **(A)** Representative images of SO&FG staining, H&E staining, and IHC for COL II and TEFM in normal control (n = 6) and OA (n = 6) cartilage; scale bar = 200 μm. **(B, C)** Quantification of the COL II– **(B)** and TEFM-positive **(C)** areas. **(D–F)** Relative mRNA expression of COL II, MMP13 and TEFM detected by qRT-PCR, normalized to GAPDH (n = 3 specimens per group). **(G)** Representative Western blot images of COL II (141 kDa), MMP13 (54 kDa), TEFM (50 kDa) and GAPDH (36 kDa). **(H–J)** Quantitative analysis of the relative protein levels normalized to GAPDH (n = 3 specimens per group). Data are presented as mean ± SD; group comparisons were performed using unpaired two-tailed Student’s t-test. *P < 0.05, **P < 0.01, ***P < 0.001.

### 3.3 TEFM knockdown alleviates IL-1β-induced chondrocyte injury and restores cell proliferation

To clarify the biological function of TEFM in chondrocytes, we stably knocked down TEFM in ATDC5 cells using lentivirus-delivered shRNA. WB and qRT-PCR confirmed efficient TEFM silencing in the sh-TEFM group, while the non-targeting sh-NC had no significant effect on basal TEFM expression (**Figures 3A–C**). Wb analysis further showed that TEFM knockdown alone did not significantly alter the basal expression levels of COL II or MMP13 (**Supplementary Figure S1**). In the IL-1β-stimulated *in vitro* OA model, TEFM knockdown markedly increased the protein expression of COL II, and simultaneously suppressed MMP13 at the protein level compared with the IL-1β + sh-NC group (**Figures 3D–F**). EdU proliferation assays showed that IL-1β stimulation profoundly inhibited ATDC5 cell proliferation, whereas TEFM silencing partially restored the proliferative capacity of IL-1β-treated cells (**Figures 3G, H**). Consistently, toluidine blue staining revealed that IL-1β treatment impaired cell morphology and proteoglycan synthesis following IL-1β treatment, both of which were ameliorated by TEFM knockdown (**Figure 3I**). IF staining further confirmed enhanced COL II fluorescence intensity and attenuated MMP13 signals in the IL-1β + sh-TEFM group relative to the IL-1β + sh-NC group (**Figures 3J–M**). Taken together, these results demonstrate that TEFM knockdown exerts protective effects against IL-1β-triggered chondrocyte damage.

**Figure 3:**
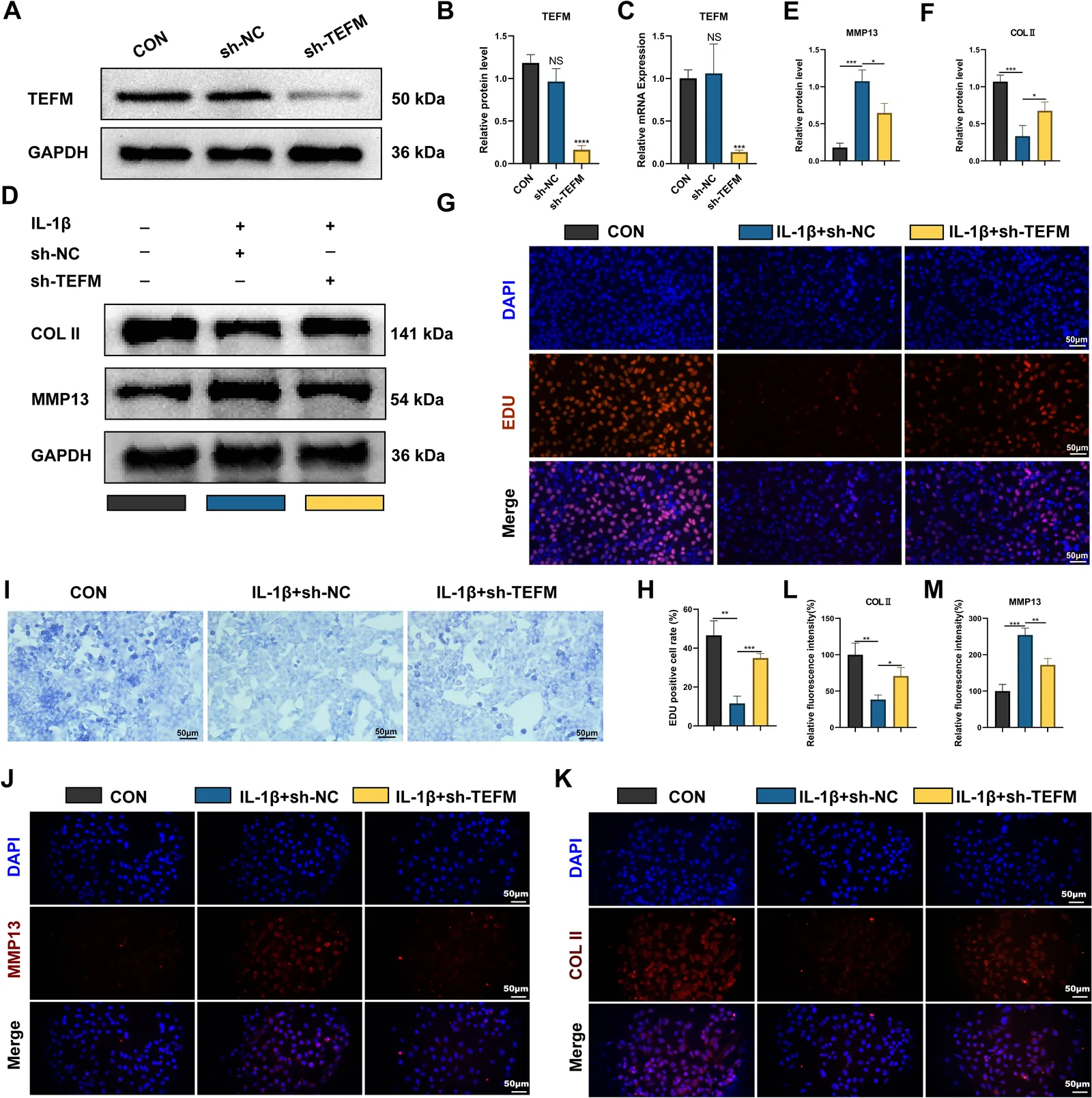
TEFM knockdown alleviates IL-1β-induced chondrocyte injury in ATDC5 cells. ATDC5 cells were left untreated (CON) or stably transduced with sh-NC or sh-TEFM, followed by 10 ng/mL IL-1β stimulation for 24 h where indicated. **(A–C)** Validation of TEFM knockdown efficiency by Western blot **(A, B)** and qRT-PCR **(C)**, normalized to GAPDH; **(D–F)** Wb images and quantitative analysis of COL II (141 kDa) and MMP13 (54 kDa) protein expression, with GAPDH (36 kDa) as the loading control; **(G, H)** EdU proliferation assay (DAPI, EdU, and merged channels; scale bar = 50 μm) and quantification of the EdU-positive cell rate; **(I)** Toluidine blue staining of proteoglycan matrix (scale bar = 50 μm); **(J–M)** IF staining (DAPI, target protein, and merged channels; scale bar = 50 μm) and quantitative analysis of COL II **(J, L)** and MMP13 **(K, M)** fluorescence intensity. Data are presented as mean ± SD from three independent experiments; comparisons between two groups were performed using unpaired two-tailed Student’s t-test. *P < 0.05, **P < 0.01, ***P < 0.001; NS, not significant.

### 3.4 TEFM knockdown reverses the IL-1β-triggered expression profile of inflammation- and matrix degradation-related genes

To further explore the molecular mechanism by which TEFM regulates chondrocyte function, RNA-seq was performed on ATDC5 cells from three groups (sh-NC, IL-1β + sh-NC, and IL-1β + sh-TEFM; n = 3 biological replicates per group). Heatmaps and volcano plots revealed that 662 genes were upregulated and 634 downregulated following IL-1β stimulation relative to the sh-NC group, predominantly involving inflammation- and matrix degradation-related genes; upon TEFM knockdown, 940 genes were downregulated versus only 255 upregulated relative to the IL-1β + sh-NC group, indicating that TEFM silencing substantially reversed the IL-1β-induced transcriptional alterations (**Figures 4A–D**). Consistent with this pattern, GO enrichment analysis showed that IL-1β-induced genes were predominantly enriched in immune response, cytokine response and stress response (**Figure 4E**), whereas the genes suppressed by TEFM knockdown were enriched in interferon-β response, cytokine-mediated signaling and response to bacterium (**Figure 4F**). KEGG pathway enrichment analysis showed that pro-inflammatory cascades, including the TNF, IL-17, NF-κB, and PI3K-Akt signaling pathways, were significantly activated following IL-1β treatment (**Figure 4G**). Upon TEFM knockdown, these pathways were also prominently enriched among the differentially expressed genes (**Figure 4H**); as this comparison was dominated by downregulated genes (940 downregulated versus 255 upregulated, **Figure 4D**), this enrichment suggests repression rather than activation of these pathways at the transcriptional level. GSEA further corroborated this bidirectional pattern: the TNF and IL-17 signaling pathways were markedly enriched in IL-1β-stimulated cells (NES = 2.04 and 2.36, adjusted P < 0.001), whereas TEFM knockdown reversed the enrichment direction (NES = −1.63 and −1.69, adjusted P < 0.05) (**Figure 4I**). Collectively, these findings indicate that TEFM knockdown alleviates inflammatory injury in chondrocytes by repressing the activation of multiple pro-inflammatory signaling pathways.

**Figure 4:**
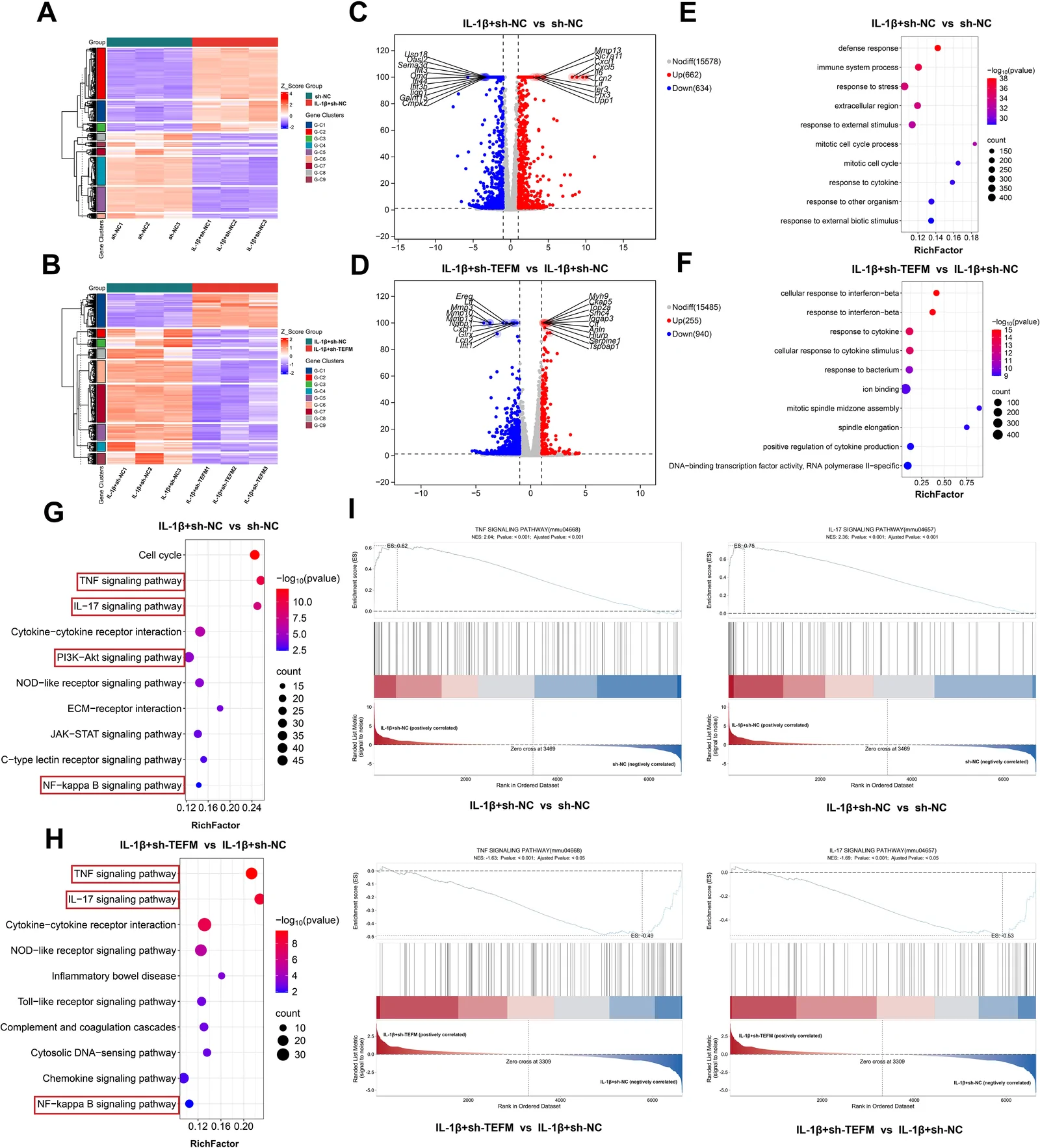
Transcriptomic analysis reveals that TEFM regulates inflammation-related signaling pathways in ATDC5 chondrocytes. RNA-seq was performed on ATDC5 cells from three groups (sh-NC, IL-1β + sh-NC, and IL-1β + sh-TEFM; n = 3 per group). **(A, C, E, G)** IL-1β + sh-NC vs sh-NC; **(B, D, F, H)** IL-1β + sh-TEFM vs IL-1β + sh-NC. **(A, B)** Heatmaps of differentially expressed genes (row-scaled Z-scores). **(C, D)** Volcano plots (|log_2_ fold change| > 1, *P* < 0.05): 662 upregulated and 634 downregulated genes upon IL-1β stimulation **(C)**; 255 upregulated and 940 downregulated upon TEFM knockdown **(D)**. **(E, F)** GO enrichment analysis (bubble size, gene count; color, −log_10_ *P* value). **(G, H)** KEGG pathway enrichment analysis. **(I)** GSEA of the TNF and IL-17 signaling pathways (NES = 2.04/2.36 upon IL-1β stimulation, adjusted P < 0.001; NES = −1.63/−1.69 upon TEFM knockdown, adjusted *P* < 0.05).

### 3.5 TEFM aggravates IL-1β-induced chondrocyte inflammatory injury via activation of the AKT/NF-κB signaling pathway

Among the pro-inflammatory pathways identified by transcriptomic enrichment, we focused on the AKT/NF-κB axis because it represents the convergent node of the TNF, IL-17 and PI3K-Akt cascades activated by IL-1β and reversed by TEFM knockdown, and because NF-κB is the master transcriptional regulator of chondrocyte catabolism directly controlling MMP13 expression. Wb analysis showed that IL-1β stimulation markedly elevated the phosphorylation of AKT and p65 in ATDC5 cells, whereas TEFM knockdown robustly suppressed p-AKT and p-p65 levels (**Figures 5A–C**). Co-expression analysis based on the GSE114007 dataset revealed a significant positive correlation between TEFM and AKT1 expression (R = 0.39, P = 0.0158) (**Figure 5D**). IF staining further illustrated that IL-1β triggered nuclear translocation of p65 from the cytoplasm, an effect markedly abrogated by TEFM silencing (**Figure 5J**).

**Figure 5:**
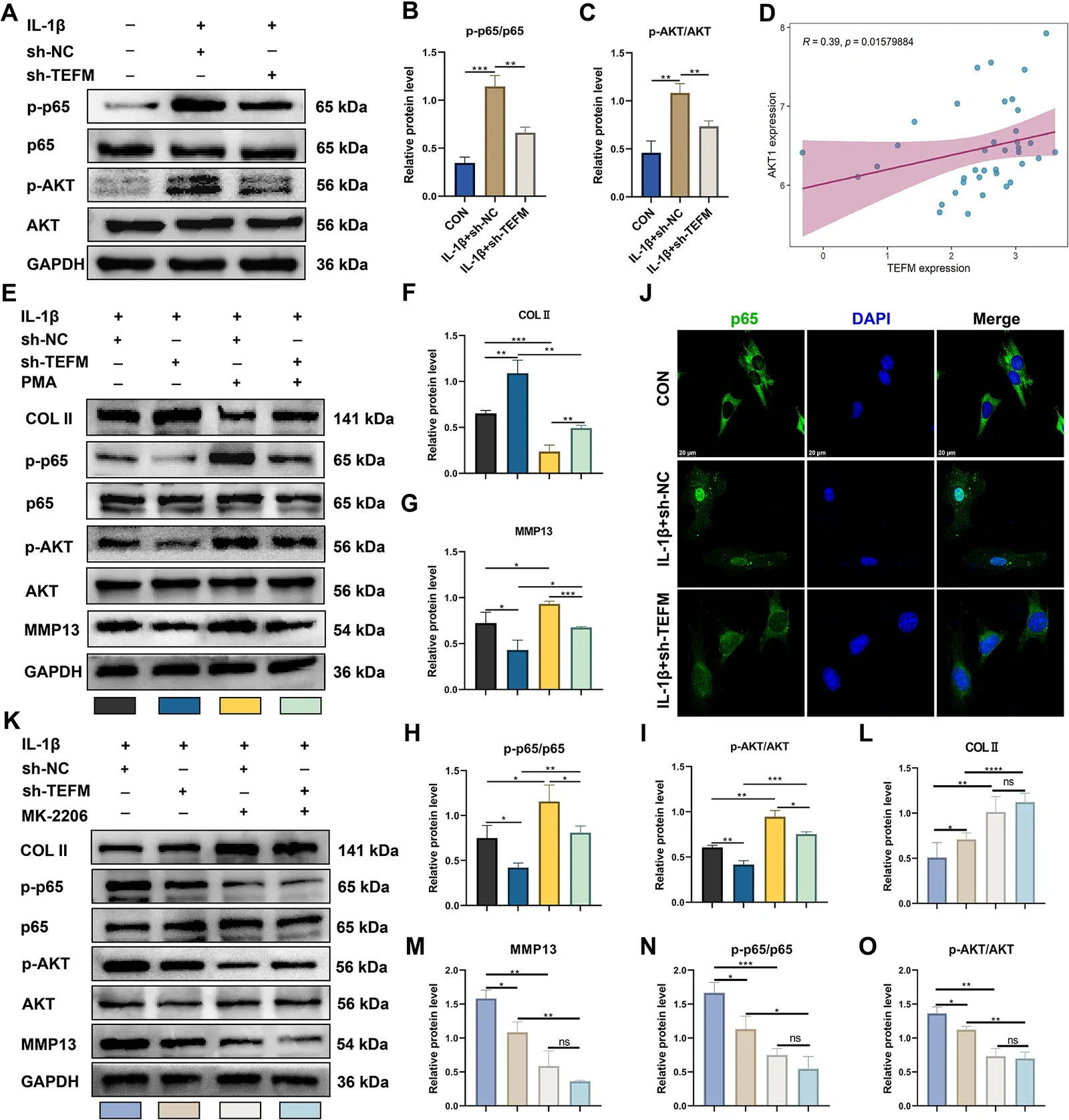
TEFM activates the AKT/NF-κB signaling pathway in ATDC5 chondrocytes. **(A–C)** Wb analysis of p-p65, p65, p-AKT, AKT and GAPDH **(A)** and quantitative analysis of the p-p65/p65 **(B)** and p-AKT/AKT **(C)** ratios in untreated control (CON), IL-1β + sh-NC, and IL-1β + sh-TEFM cells. **(D)** Co-expression analysis of TEFM and AKT1 in the GSE114007 dataset (Pearson correlation). **(E–I)** Wb **(E)** and quantitative analysis of COL II **(F)**, MMP13 **(G)**, p-p65/p65 **(H)**, and p-AKT/AKT **(I)** in IL-1β-stimulated TEFM-knockdown cells treated with or without PMA (100 nM), an NF-κB pathway activator. **(J)** Immunofluorescence staining of p65 (green), DAPI (blue), and merged images showing p65 nuclear translocation; scale bar = 20 μm. **(K–O)** Western blot **(K)** and quantitative analysis of COL II **(L)**, MMP13 **(M)**, p-p65/p65 **(N)**, and p-AKT/AKT **(O)** in IL-1β-stimulated cells treated with TEFM knockdown with or without MK-2206 (10 μM), a selective AKT inhibitor. Data are presented as mean ± SD from three independent experiments; comparisons between two groups were performed using unpaired two-tailed Student’s t-test. *P < 0.05, **P < 0.01, ***P < 0.001; NS, not significant.

To determine whether AKT acts upstream of NF-κB in this cascade, cells were pretreated with MK-2206 (10 μM, 2 h), a selective allosteric AKT inhibitor. Compared with the IL-1β + sh-NC group, MK-2206 treatment markedly reduced IL-1β-induced p-AKT and p-p65 levels and reproduced the effects of TEFM knockdown, namely decreased MMP13 and restored COL II expression (**Figures 5K–O**). Notably, no additive effect on p-AKT or p-p65 was observed when MK-2206 was combined with TEFM knockdown (**Figures 5N, O**), indicating that TEFM and AKT act within the same linear pathway. This, together with the reduction of p-AKT by TEFM silencing (**Figure 5C**) and the suppression of p-p65 by AKT inhibition, places AKT downstream of TEFM and upstream of NF-κB in this cascade.

Furthermore, rescue experiments were performed using PMA, an agonist of the NF-κB pathway. PMA treatment partially reversed both the upregulation of COL II and the repression of MMP13, p-AKT and p-p65 induced by TEFM knockdown (**Figures 5E–I**). Collectively, these data confirm that TEFM mediates IL-1β-evoked chondrocyte inflammatory damage through activation of the AKT/NF-κB signaling pathway.

### 3.6 TEFM activates the AKT/NF-κB pathway by promoting ROS production

Given that enrichment analyses indicated a close association between TEFM and mitochondrial function, we next assessed intracellular ROS levels and mitochondrial status. DCFH-DA staining revealed that IL-1β stimulation markedly elevated intracellular ROS levels, whereas TEFM knockdown significantly suppressed ROS accumulation (**Figures 6A, C**). Consistently, MitoSOX staining showed that IL-1β-induced mitochondrial superoxide production was attenuated by TEFM silencing (**Figures 6B, D**). TMRM staining further demonstrated that IL-1β triggered mitochondrial membrane potential dissipation, which was partially restored upon TEFM knockdown (**Figure 6E**). TEM revealed swollen mitochondria with disrupted cristae in IL-1β-treated cells, while TEFM-silenced cells retained relatively intact mitochondrial ultrastructure (**Figure 6F**). These results indicate that TEFM knockdown alleviates IL-1β-induced mitochondrial oxidative stress.

**Figure 6:**
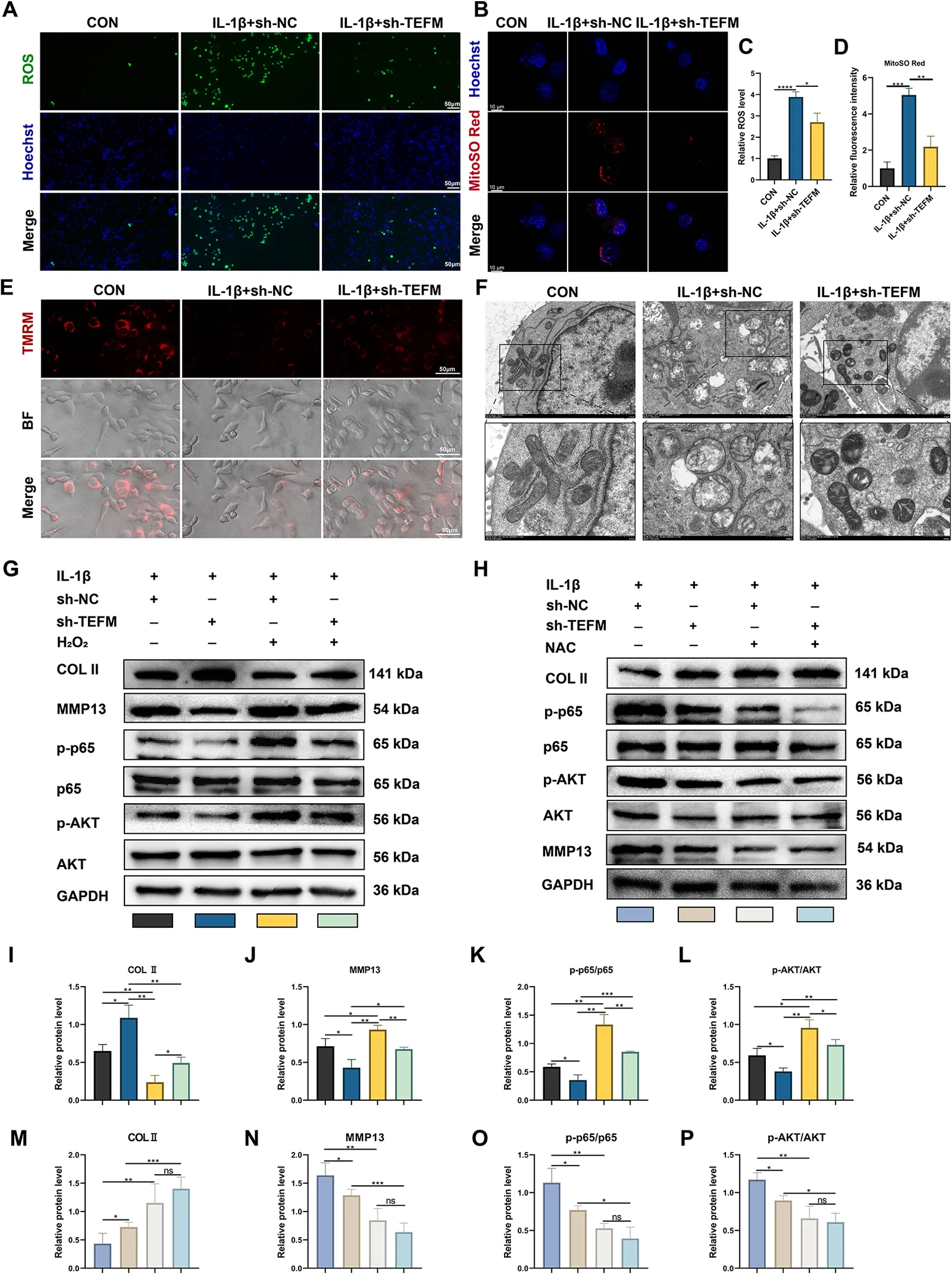
TEFM activates the AKT/NF-κB pathway in a ROS-dependent manner. **(A)** Representative images of intracellular ROS detected by the DCFH-DA probe (green) with Hoechst 33342 nuclear counterstaining and merged views; scale bar = 50 μm. **(B)** MitoSOX Red staining (red) showing mitochondrial superoxide levels, with Hoechst 33342 nuclear counterstaining; scale bar = 10 μm. **(C, D)** Quantification of relative DCFH-DA **(C)** and MitoSOX Red **(D)** fluorescence intensity. **(E)** TMRM staining (red) showing mitochondrial membrane potential, with bright field (BF) images; scale bar = 50 μm. **(F)** Representative TEM images of mitochondrial ultrastructure; lower panels show magnified views of the boxed regions. **(G)** Wb analysis of COL II, MMP13, p-p65, p65, p-AKT, and AKT in IL-1β-stimulated TEFM-knockdown cells treated with or without H_2_O_2_ (200 μM). **(H)** Wb analysis of COL II, MMP13, p-p65, p65, p-AKT, and AKT in IL-1β-stimulated cells treated with or without the ROS scavenger NAC (5 mM). **(I–L)** Quantitative analysis of COL II **(I)**, MMP13 **(J)**, p-p65/p65 **(K)**, and p-AKT/AKT **(L)** from the H_2_O_2_ rescue experiment. **(M–P)** Quantitative analysis of COL II **(M)**, MMP13 **(N)**, p-p65/p65 **(O)**, and p-AKT/AKT **(P)** from the NAC experiment. Data are presented as mean ± SD from three independent experiments; comparisons between two groups were performed using unpaired two-tailed Student’s t-test. *P < 0.05, **P < 0.01, ***P < 0.001, ****P < 0.0001; NS, not significant.

To verify the mediating role of ROS in TEFM-dependent activation of the AKT/NF-κB cascade, rescue and scavenging assays were performed. Exogenous H_2_O_2_ (200 μM) substantially reversed the inhibitory effects of TEFM knockdown on p-AKT and p-p65 and aggravated IL-1β-induced chondrocyte injury, as evidenced by reduced COL II and increased MMP13 (**Figures 6G, I–L**). Conversely, pretreatment with the ROS scavenger NAC (5 mM) in IL-1β-stimulated cells mimicked the effects of TEFM silencing: p-AKT and p-p65 levels were markedly reduced, COL II was restored, and MMP13 was suppressed (**Figures 6H, M–P**). Taken together, these data demonstrate that TEFM drives chondrocyte injury by promoting ROS accumulation, which in turn activates the AKT/NF-κB signaling pathway.

### 3.7 Intra-articular injection of AAV-sh-TEFM alleviates OA progression in DMM mice

To verify the *in vivo* function of TEFM, a surgically induced DMM OA mouse model was established. One week after surgery, adeno-associated virus carrying AAV-sh-TEFM or AAV-sh-NC was injected into the knee joint cavity (**Figure 7A**). Micro-CT analysis performed at week 9 postoperatively revealed that compared with the Sham group, the DMM+AAV-sh-NC group exhibited significantly reduced BV/TV, Tb.N and Tb.Th, alongside markedly elevated Tb.Sp in the subchondral bone of the tibial plateau. Intra-articular administration of AAV-sh-TEFM substantially reversed such trabecular microstructural deterioration (**Figures 7B–F**).

**Figure 7:**
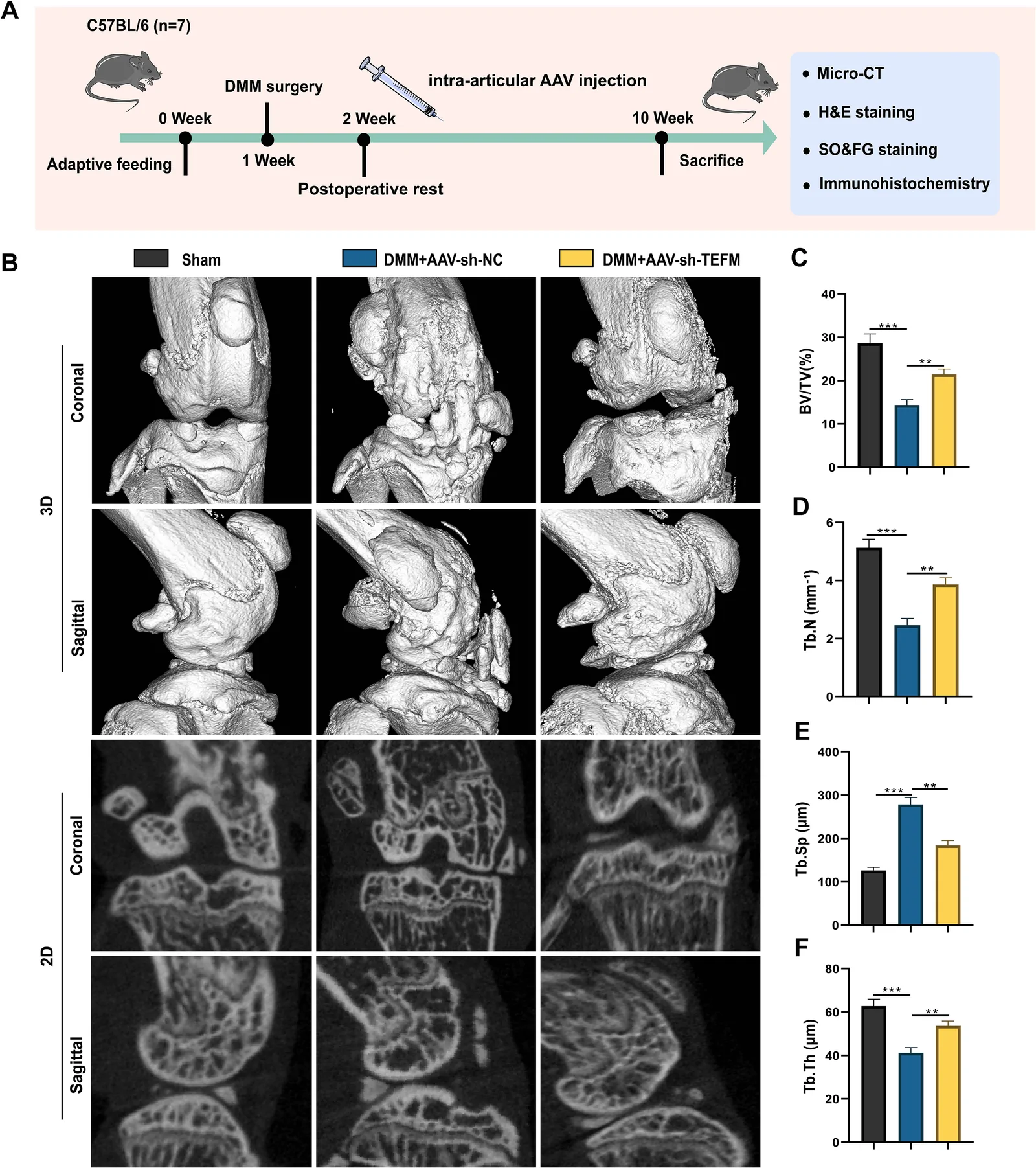
TEFM knockdown alleviates subchondral bone microstructural deterioration in DMM mice. **(A)** Schematic workflow of the *in vivo* experiment: DMM surgery was performed at week 1, intra-articular injection of AAV9-sh-NC or AAV9-sh-TEFM (10 μL, 1 × 10¹² vg/mL) at week 2, and knee joints were harvested at week 10 for Micro-CT and histological analyses (n = 7 mice per group). **(B)** Three-dimensional reconstructed coronal and sagittal views (upper and middle rows) and two-dimensional images of the tibial subchondral bone region of interest (lower row). **(C–F)** Quantitative analysis of bone microarchitecture parameters: bone volume fraction (BV/TV, **(C)**), trabecular number (Tb.N, **(D)**), trabecular separation (Tb.Sp, **(E)**), and trabecular thickness (Tb.Th, F). Data are presented as mean ± SD (n = 7); comparisons between two groups were performed using unpaired two-tailed Student’s t-test. *P < 0.05, **P < 0.01, ***P < 0.001.

Histological assessment further demonstrated that the DMM+AAV-sh-NC group displayed rough articular cartilage surfaces, thinner cartilage layers and severe proteoglycan loss, accompanied by drastically increased OARSI scores. In contrast, the DMM+AAV-sh-TEFM group retained relatively intact cartilage architecture with significantly lower OARSI scores (**Figures 8A, B**). IHC staining showed that cartilage tissues from the DMM+AAV-sh-NC group presented pronounced downregulation of COL II and upregulation of MMP13, p-AKT and p-p65; TEFM knockdown significantly rescued these abnormal expression patterns (**Figures 8A, C–F**). Collectively, these *in vivo* findings demonstrate that TEFM silencing exerts chondroprotective effects and effectively retards DMM-triggered OA progression via repressing activation of the AKT/NF-κB signaling pathway.

**Figure 8:**
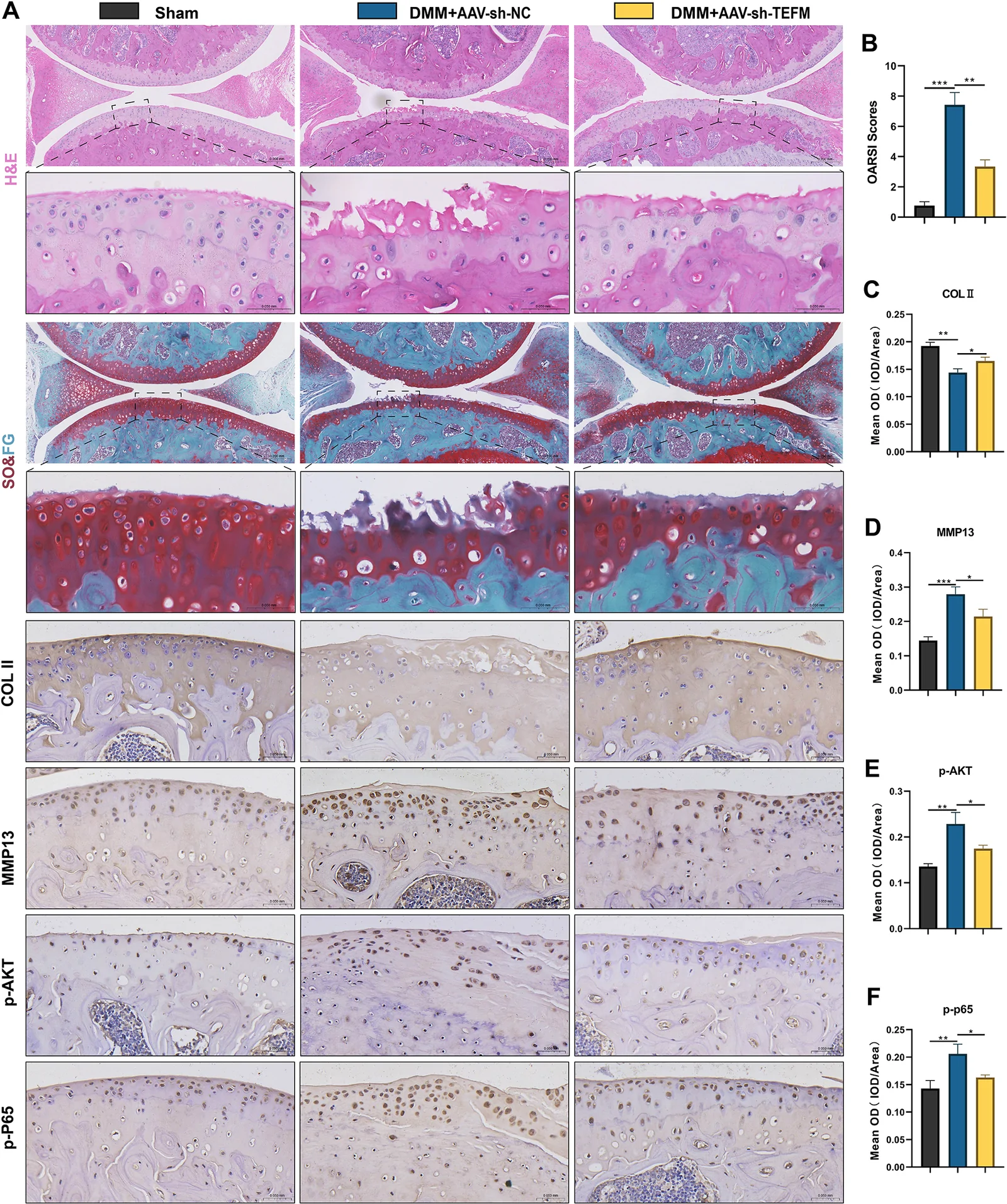
TEFM knockdown mitigates articular cartilage degeneration in DMM mice. **(A)** Representative images of H&E staining, SO&FG staining, and IHC for COL II, MMP13, p-AKT, and p-p65 in knee articular cartilage; upper panels show low-magnification views (boxed regions, scale bar = 200 μm) and lower panels show magnified views of the boxed areas (scale bar = 50 μm). **(B)** OARSI histological scores of cartilage degeneration. **(C–F)** Quantitative analysis of the mean optical density (MOD) of COL II **(C)**, MMP13 **(D)**, p-AKT **(E)**, and p-p65 **(F)** IHC staining. Data are presented as mean ± SD (n = 7 mice per group); comparisons between two groups were performed using unpaired two-tailed Student’s t-test. *P < 0.05, **P < 0.01, ***P < 0.001.

## 4 Discussion

OA is a complex multifactorial disorder characterized by progressive degeneration of articular cartilage. Its pathogenesis involves multiple layers including abnormal mechanical stress, inflammatory responses, extracellular matrix degradation and disrupted chondrocyte metabolism (3–5). In recent years, the roles of mitochondrial dysfunction and oxidative stress have been increasingly recognized as key contributors to OA progression; nevertheless, the specific functions of mitochondrial transcriptional regulators in OA remain largely unexplored. The present study identifies mitochondrial transcription elongation factor TEFM as a previously uncharacterized regulator of OA initiation and progression. Mechanistically, TEFM exacerbates inflammatory injury and matrix degradation in chondrocytes by boosting ROS accumulation, which in turn activates the AKT/NF-κB signaling axis. These findings provide new insights into the molecular pathological mechanisms of OA from the perspective of mitochondrial transcriptional regulation.

Consistent results from bioinformatic screening, clinical specimen validation and *in vitro* & *in vivo* functional experiments collectively confirm that TEFM is markedly upregulated in OA cartilage and IL-1β-injured chondrocytes. As a core component governing mitochondrial gene transcription, TEFM interacts with mitochondrial RNA polymerase to guarantee intact expression of oxidative phosphorylation complexes, which is indispensable for sustaining mitochondrial respiratory function and cellular energy metabolism (9, 10, 12). Mounting evidence has linked impaired mitochondrial homeostasis to OA pathogenesis: mitochondrial dysfunction suppresses oxidative phosphorylation and ATP production, rendering chondrocytes incapable of maintaining balanced matrix anabolism and cartilage homeostasis (20–22). Meanwhile, mitochondria-derived ROS (mtROS) not only directly inflict cellular damage but also amplify inflammatory and catabolic cascades (23, 24). Recent investigations have highlighted that disrupted mitochondrial quality control—encompassing mitochondrial dynamics, biogenesis, antioxidant defense and mitophagy—is tightly associated with OA onset and advancement (25–28). Under physiological conditions, TEFM expression is tightly restrained to preserve mitochondrial homeostasis; under pathological circumstances, aberrantly elevated TEFM may disrupt the balance between mitochondrial transcription and energy demand, contributing to the development of multiple diseases (11, 13–15). Prior oncological research reported excessive TEFM expression in uterine corpus endometrial carcinoma (UCEC): TEFM overexpression impairs mitochondrial function, diminishes mitochondrial membrane potential, triggers ROS-mediated inflammatory pathway activation and enhances autophagy, ultimately driving UCEC proliferation and metastasis (15). Another study documented elevated TEFM levels in hepatocellular carcinoma, where TEFM upregulation facilitates tumor growth and metastasis by increasing ROS production and activating the ERK signaling cascade (14). In our work, high TEFM expression strongly correlates with reduced COL II, elevated MMP13 and aggravated cartilage degeneration in OA cartilage, implying TEFM as a prospective biomarker for OA progression. Mendelian randomization analysis further substantiates the causal link between TEFM and OA susceptibility, reinforcing the credibility of TEFM as a therapeutic target.

Functionally, *in vitro* assays reveal that TEFM knockdown significantly elevates COL II abundance, suppresses MMP13 expression, and partially restores proliferative capacity suppressed by IL-1β in chondrocytes. COL II constitutes the primary structural component of the cartilage extracellular matrix, and diminished COL II synthesis coupled with MMP13-mediated matrix breakdown represents the central event underlying OA cartilage deterioration. Transcriptome sequencing further demonstrates that TEFM silencing substantially reverses the transcriptional signature of inflammation and matrix degradation induced by IL-1β, while inhibiting the activation of multiple pro-inflammatory pathways including TNF, IL-17, NF-κB and PI3K-Akt signaling pathways. Taken together with the mitochondrial findings described above, these data indicate that TEFM serves not only as a regulator of mitochondrial function but also disrupts the anabolic-catabolic equilibrium of chondrocytes via downstream signaling cascades. Notably, in the absence of IL-1β stimulation, TEFM knockdown did not significantly alter basal COL II or MMP13 expression, indicating that the protective effect of TEFM silencing is specific to pathological inflammatory conditions rather than reflecting a general disruption of basal chondrocyte metabolism.

Mechanistically, this study delineates the core function of the TEFM–ROS–AKT/NF-κB axis in mediating chondrocyte damage during OA. Co-expression analysis based on the GSE114007 dataset revealed a significant positive correlation between TEFM and AKT1 expression. Wb and IF assays verify that IL-1β stimulation robustly induces phosphorylation of AKT and p65 and accelerates p65 nuclear translocation, all of which are effectively abrogated by TEFM knockdown. Critically, TEFM silencing markedly alleviates IL-1β-triggered intracellular and mitochondrial ROS overproduction, preserves mitochondrial ultrastructure, and restores mitochondrial membrane potential, suggesting that TEFM impairs mitochondrial homeostasis to drive excessive ROS generation. This direction may seem counterintuitive given TEFM’s canonical role in supporting mitochondrial transcription. We propose that pathological TEFM overexpression uncouples mtDNA transcription from respiratory demand, thereby increasing electron leakage and ROS, whereas partial knockdown restores this balance—consistent with the reduced oxidative stress observed upon TEFM silencing.

Previous studies have likewise confirmed that TEFM upregulation provokes mitochondrial dysfunction, depolarizes mitochondrial membranes and augments ROS synthesis (14, 15). The functional hierarchy within this cascade was further resolved by pharmacological epistasis: the AKT inhibitor MK-2206 phenocopied the effects of TEFM knockdown with no additive effect on pathway phosphorylation, placing AKT downstream of TEFM and upstream of NF-κB, while NF-κB activation by PMA partially reversed the protective effects of TEFM silencing. Rescue experiments with exogenous H_2_O_2_ and ROS scavenging with NAC further establish ROS as a critical upstream mediator of TEFM-driven AKT/NF-κB activation—the former demonstrating sufficiency, the latter necessity. Mechanistically, ROS can activate AKT through oxidative modification of upstream regulators such as PTEN, and activates the IKK complex to accelerate IκB degradation, thereby liberating NF-κB to translocate into the nucleus and initiate transcription of downstream pro-inflammatory cytokines and MMPs. Accordingly, TEFM mediates a core pathogenic cascade consisting of dysregulated mitochondrial transcription, ROS burst and subsequent AKT/NF-κB activation, serving as a molecular bridge linking inflammation and metabolic disturbance in OA progression.

For *in vivo* validation, we adopted the classic DMM mouse OA model and performed local gene intervention via intra-articular injection of AAV-sh-TEFM adeno-associated virus. Consistently, Micro-CT and histological analyses demonstrate that TEFM knockdown remarkably alleviates DMM-induced microstructural destruction of subchondral bone, mitigates the severity of articular cartilage degeneration, and represses the expression of MMP13, p-AKT and p-p65 in cartilage tissues. It is noteworthy that the DMM + AAV-sh-NC group exhibited decreased bone volume fraction, trabecular number and trabecular thickness, rather than the subchondral bone sclerosis often reported in late-stage OA. This apparent discrepancy likely reflects the time-dependent nature of subchondral bone remodeling following DMM surgery: trabecular bone loss and enhanced resorption predominate in the early-to-middle phase (approximately 4–8 weeks post-surgery), whereas osteoblastic sclerosis and osteophyte formation emerge in the late phase (beyond 12 weeks) (29, 30). Our harvest time point (week 9) therefore captures the resorptive phase of bone remodeling, and the measured parameters (BV/TV, Tb.N, Tb.Th) primarily reflect trabecular bone in the epiphyseal region rather than the subchondral plate itself. Future studies employing longer observation periods and dynamic histomorphometry will be necessary to determine whether TEFM silencing likewise mitigates late-stage subchondral sclerosis. These *in vivo* findings not only corroborate the physiological relevance of our *in vitro* observations but also highlight the therapeutic potential of local intra-articular TEFM targeting for OA treatment. As a minimally invasive administration route, intra-articular injection enables concentrated drug retention within the joint cavity and minimizes systemic adverse effects, laying a practical foundation for the future development of TEFM-targeted OA therapies.

Several limitations of this study should be acknowledged. First, the MR analysis was based on only five cis-eQTL SNPs; this small instrument set limits the precision of MR-Egger and weighted median estimates, and the resulting causal inference—rather than mere correlation—should be considered suggestive until validated in cartilage-derived eQTL cohorts, given that whole-blood eQTLs may not reflect chondrocyte-specific regulation. Second, all *in vitro* functional experiments were performed using the ATDC5 chondrocyte cell line; follow-up studies incorporating primary human or mouse chondrocytes are required to validate the generalizability of our conclusions. Third, only male mice were utilized for *in vivo* experiments. OA exhibits prominent gender disparities, and estrogen exerts well-established regulatory effects on mitochondrial function and oxidative stress. Future investigations should include female animals to dissect how sex modulates the pathological impacts of TEFM. Fourth, although TEFM is a mitochondrial transcription factor, we did not directly measure its transcriptional output (mitochondrial RNA levels), respiratory chain complex assembly/activity, or mitochondrial DNA (mtDNA) copy number; consequently, the molecular events linking TEFM to ROS overproduction remain incompletely defined and warrant dedicated mitochondrial functional assays in future studies. Fifth, our mechanistic focus is restricted to the AKT/NF-κB pathway. Transcriptomic enrichment hints at potential involvement of MAPK signaling and autophagy in TEFM-mediated cartilage damage, and the cross-talk networks among these distinct pathways remain to be elucidated; NF-κB-specific chemical inhibition (e.g., BAY 11-7082) should also be included in future epistasis experiments. Sixth, our quantitative analyses of MMP13 were based on the ~54 kDa latent/pro-form, because the active ~45 kDa form was faint and not consistently quantifiable across all biological replicates. Future studies using MMP13 activity assays (e.g., fluorogenic substrate assays) will be necessary to confirm the changes in active MMP13. Finally, this study relied solely on loss-of-function strategies. Gain-of-function validation by TEFM overexpression, together with subsequent screening of small-molecule TEFM modulators, remains to be performed.

In conclusion, the present study identifies aberrant TEFM upregulation in OA cartilage and defines its pathogenic role in promoting cartilage degeneration through the ROS–AKT/NF-κB signaling axis. TEFM may therefore represent a candidate molecular marker of OA progression and a potential therapeutic target for slowing OA progression by modulating mitochondrial function and inflammatory responses.

## 5 Conclusions

This study confirms that TEFM is aberrantly upregulated in osteoarthritic cartilage. TEFM aggravates chondrocyte inflammatory injury and extracellular matrix degradation by facilitating ROS overproduction to activate the AKT/NF-κB signaling pathway (**Figure 9**). Targeted knockdown of TEFM effectively alleviates OA progression both *in vitro* and *in vivo*, indicating that the TEFM–ROS–AKT/NF-κB axis represents a promising therapeutic target for OA treatment.

**Figure 9:**
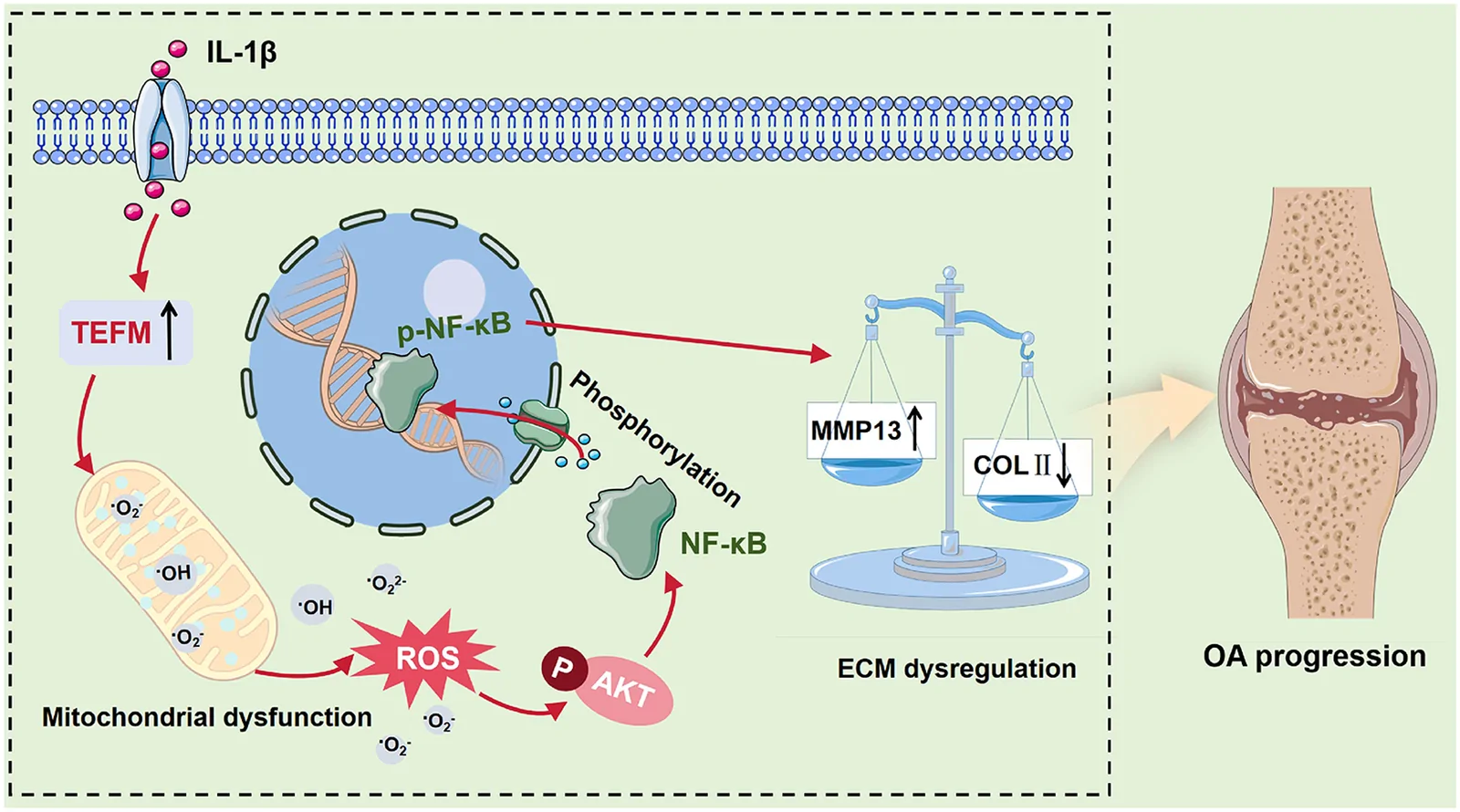
Schematic diagram illustrating the proposed mechanism by which TEFM exacerbates OA via the ROS–AKT/NF-κB signaling axis. In chondrocytes, IL-1β stimulation upregulates TEFM expression, which impairs mitochondrial homeostasis and promotes excessive ROS generation. Accumulated ROS activates AKT, leading to phosphorylation and nuclear translocation of NF-κB, which in turn upregulates MMP13 and downregulates COL II, resulting in extracellular matrix (ECM) dysregulation and eventual OA progression.

## Data Availability

The datasets presented in this study can be found in online repositories. The names of the repository/repositories and accession number(s) can be found in the article/**Supplementary Material**.

## References

[1] Gbd 2021 Osteoarthritis Collaborators . Global, regional, and national burden of osteoarthritis, 1990-2020 and projections to 2050: A systematic analysis for the global burden of disease study 2021. Lancet Rheumatol. (2023) 5:e508–22. doi: 10.1016/S2665-9913(23)00163-7 PMC10477960 37675071

[2] Cho Y Jeong S Kim H Kang D Lee J Kang SB . Disease-modifying therapeutic strategies in osteoarthritis: Current status and future directions. Exp Mol Med. (2021) 53:1689–96. doi: 10.1038/s12276-021-00710-y 34848838 PMC8640059

[3] He Y Li Z Alexander PG Ocasio-Nieves BD Yocum L Lin H . Pathogenesis of osteoarthritis: Risk factors, regulatory pathways in chondrocytes, and experimental models. Biology. (2020) 9:194. doi: 10.3390/biology9080194 32751156 PMC7464998

[4] Coaccioli S Sarzi-Puttini P Zis P Rinonapoli G Varrassi G . Osteoarthritis: New insight on its pathophysiology. J Clin Med. (2022) 11:6013. doi: 10.3390/jcm11206013 36294334 PMC9604603

[5] Guo P Alhaskawi A Adel Abdo Moqbel S Pan Z . Recent development of mitochondrial metabolism and dysfunction in osteoarthritis. Front Pharmacol. (2025) 16:1538662. doi: 10.3389/fphar.2025.1538662 40017603 PMC11865096

[6] Zhang M Wu J Cai K Liu Y Lu B Zhang J . From dysfunction to healing: Advances in mitochondrial therapy for osteoarthritis. J Transl Med. (2024) 22:1013. doi: 10.1186/s12967-024-05799-z 39529128 PMC11552139

[7] Zheng L Zhang Z Sheng P Mobasheri A . The role of metabolism in chondrocyte dysfunction and the progression of osteoarthritis. Ageing Res Rev. (2021) 66:101249. doi: 10.1016/j.arr.2020.101249 33383189

[8] Hillen HS Temiakov D Cramer P . Structural basis of mitochondrial transcription. Nat Struct Mol Biol. (2018) 25:754–65. doi: 10.1107/s2053273318094585 30190598 PMC6583890

[9] Jiang S Koolmeister C Misic J Siira S Kühl I Silva Ramos E . TEFM regulates both transcription elongation and RNA processing in mitochondria. EMBO Rep. (2019) 20:e48101. doi: 10.15252/embr.201948101 31036713 PMC6549021

[10] Posse V Shahzad S Falkenberg M Hällberg BM Gustafsson CM . TEFM is a potent stimulator of mitochondrial transcription elongation *in vitro*. Nucleic Acids Res. (2015) 43:2615–24. doi: 10.1093/nar/gkv105 25690892 PMC4357710

[11] Van Haute L O’Connor E Díaz-Maldonado H Munro B Polavarapu K Hock DH . TEFM variants impair mitochondrial transcription causing childhood-onset neurological disease. Nat Commun. (2023) 14:1009. doi: 10.1038/s41467-023-36277-7 36823193 PMC9950373

[12] Minczuk M He J Duch AM Ettema TJ Chlebowski A Dzionek K . TEFM (c17orf42) is necessary for transcription of human mtDNA. Nucleic Acids Res. (2011) 39:4284–99. doi: 10.1093/nar/gkq1224 21278163 PMC3105396

[13] Hu W Yang J Hu K Luo G Chen Z Lu Z . Identification of TEFM as a potential therapeutic target for LUAD treatment. J Transl Med. (2024) 22:692. doi: 10.1186/s12967-024-05483-2 39075464 PMC11288054

[14] Wan L Wang Y Zhang Z Wang J Niu M Wu Y . Elevated TEFM expression promotes growth and metastasis through activation of ROS/ERK signaling in hepatocellular carcinoma. Cell Death Dis. (2021) 12:325. doi: 10.1038/s41419-021-03618-7 33771980 PMC7997956

[15] Lei J Zhu Q Guo J Chen J Qi L Cui M . TEFM facilitates uterine corpus endometrial carcinoma progression by activating ROS-NFκB pathway. J Transl Med. (2024) 22:1151. doi: 10.1186/s12967-024-05833-0 39731053 PMC11673371

[16] Bolduc JA Collins JA Loeser RF . Reactive oxygen species, aging and articular cartilage homeostasis. Free Radic Biol Med. (2019) 132:73–82. doi: 10.1016/j.freeradbiomed.2018.08.038 30176344 PMC6342625

[17] Arra M Swarnkar G Ke K Otero JE Ying J Duan X . LDHA-mediated ROS generation in chondrocytes is a potential therapeutic target for osteoarthritis. Nat Commun. (2020) 11:3427. doi: 10.1038/s41467-020-17242-0 32647171 PMC7347613

[18] Catheline SE Bell RD Oluoch LS James MN Escalera-Rivera K Maynard RD . IKKβ-NF-κB signaling in adult chondrocytes promotes the onset of age-related osteoarthritis in mice. Sci Signaling. (2021) 14:eabf3535. doi: 10.1126/scisignal.abf3535 34546791 PMC8734558

[19] Huang W Hu W Chen J Yuan D . Identification of novel protein biomarkers for knee osteoarthritis by integrating human plasma proteome: Evidence from mendelian randomization and preliminary *in vitro* investigation. Front Med (Lausanne). (2026) 13:1745114. doi: 10.3389/fmed.2026.1745114 42100251 PMC13143750

[20] Wu X Liyanage C Plan M Stark T McCubbin T Barrero RA . Dysregulated energy metabolism impairs chondrocyte function in osteoarthritis. Osteoarthritis Cartilage. (2023) 31:613–26. doi: 10.1016/j.joca.2022.11.004 36410637

[21] Zhang Y Hou M Liu Y Liu T Chen X Shi Q . Recharge of chondrocyte mitochondria by sustained release of melatonin protects cartilage matrix homeostasis in osteoarthritis. J Pineal Res. (2022) 73:e12815. doi: 10.1111/jpi.12815 35726138

[22] Tan S Sun Y Li S Wu H Ding Y . The impact of mitochondrial dysfunction on osteoarthritis cartilage: Current insights and emerging mitochondria-targeted therapies. Bone Res. (2025) 13:77. doi: 10.1038/s41413-025-00460-x 40887466 PMC12399774

[23] Mukherjee A Ghosh KK Chakrabortty S Gulyás B Padmanabhan P Ball WB . Mitochondrial reactive oxygen species in infection and immunity. Biomolecules. (2024) 14:670. doi: 10.3390/biom14060670 38927073 PMC11202257

[24] Zhao M Wang Y Li L Liu S Wang C Yuan Y . Mitochondrial ROS promote mitochondrial dysfunction and inflammation in ischemic acute kidney injury by disrupting TFAM-mediated mtDNA maintenance. Theranostics. (2021) 11:1845–63. doi: 10.7150/thno.50905 33408785 PMC7778599

[25] Cheung C Tu S Feng Y Wan C Ai H Chen Z . Mitochondrial quality control dysfunction in osteoarthritis: Mechanisms, therapeutic strategies & future prospects. Arch Gerontol Geriatr. (2024) 125:105522. doi: 10.1016/j.archger.2024.105522 38861889

[26] Liu D Cai ZJ Yang YT Lu WH Pan LY Xiao WF . Mitochondrial quality control in cartilage damage and osteoarthritis: New insights and potential therapeutic targets. Osteoarthritis Cartilage. (2022) 30:395–405. doi: 10.1016/j.joca.2021.10.009 34715366

[27] Zanfardino P Amati A Perrone M Petruzzella V . The balance of MFN2 and OPA1 in mitochondrial dynamics, cellular homeostasis, and disease. Biomolecules. (2025) 15:433. doi: 10.3390/biom15030433 40149969 PMC11940761

[28] Sun K Jing X Guo J Yao X Guo F . Mitophagy in degenerative joint diseases. Autophagy. (2021) 17:2082–92. doi: 10.1080/15548627.2020.1822097 PMC8496714 32967533

[29] Burr DB Gallant MA . Bone remodelling in osteoarthritis. Nat Rev Rheumatol. (2012) 8:665–73. doi: 10.1038/nrrheum.2012.130 22868925

[30] Fang H Huang L Welch I Norley C Holdsworth DW Beier F . Early changes of articular cartilage and subchondral bone in the DMM mouse model of osteoarthritis. Sci Rep. (2018) 8:2855. doi: 10.1038/s41598-018-21184-5 29434267 PMC5809364

