# Supplementary material for: TEFM drives cartilage degeneration in osteoarthritis via the ROS-AKT/NF-κB signaling axis
Source: Front Immunol. 2026 Oct 7;17:1961836. doi: 10.3389/fimmu.2026.1961836 (PMC13685904; doi:10.3389/fimmu.2026.1961836)

Figure 2G WB data

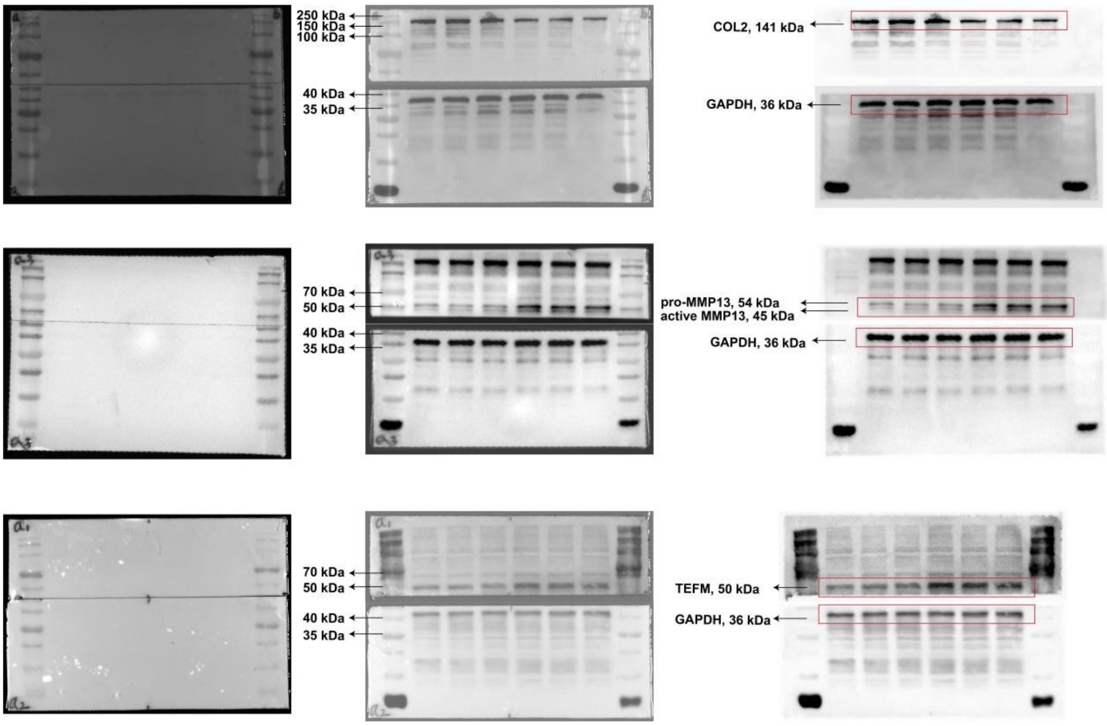

Figure 3 WB data

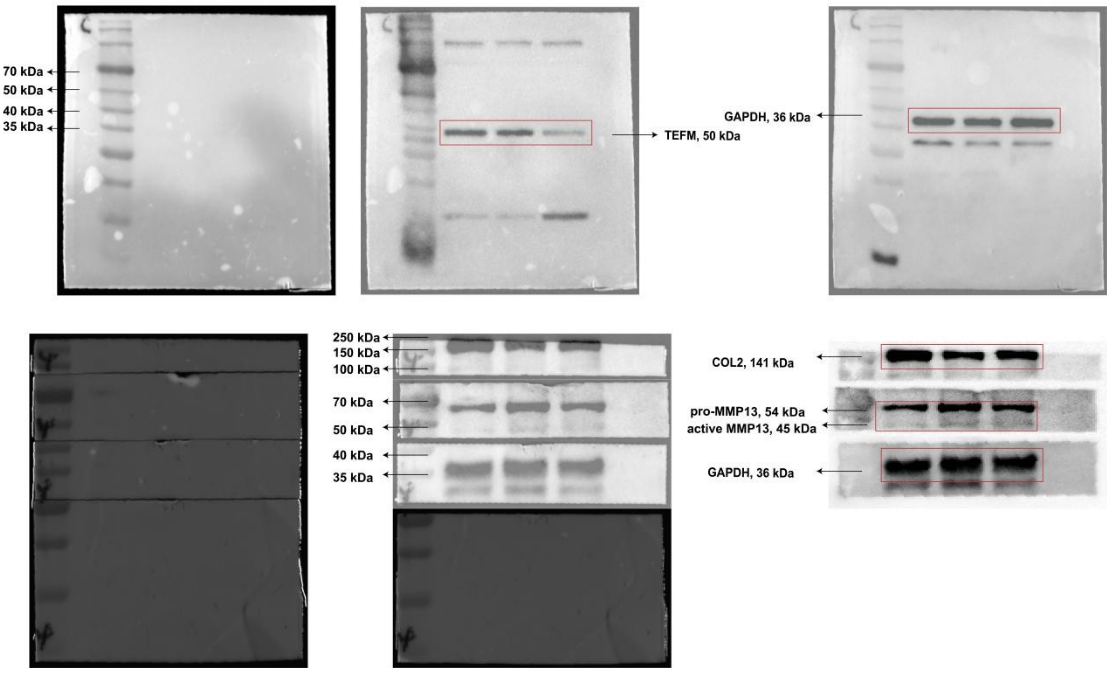

Figure 5A WB data

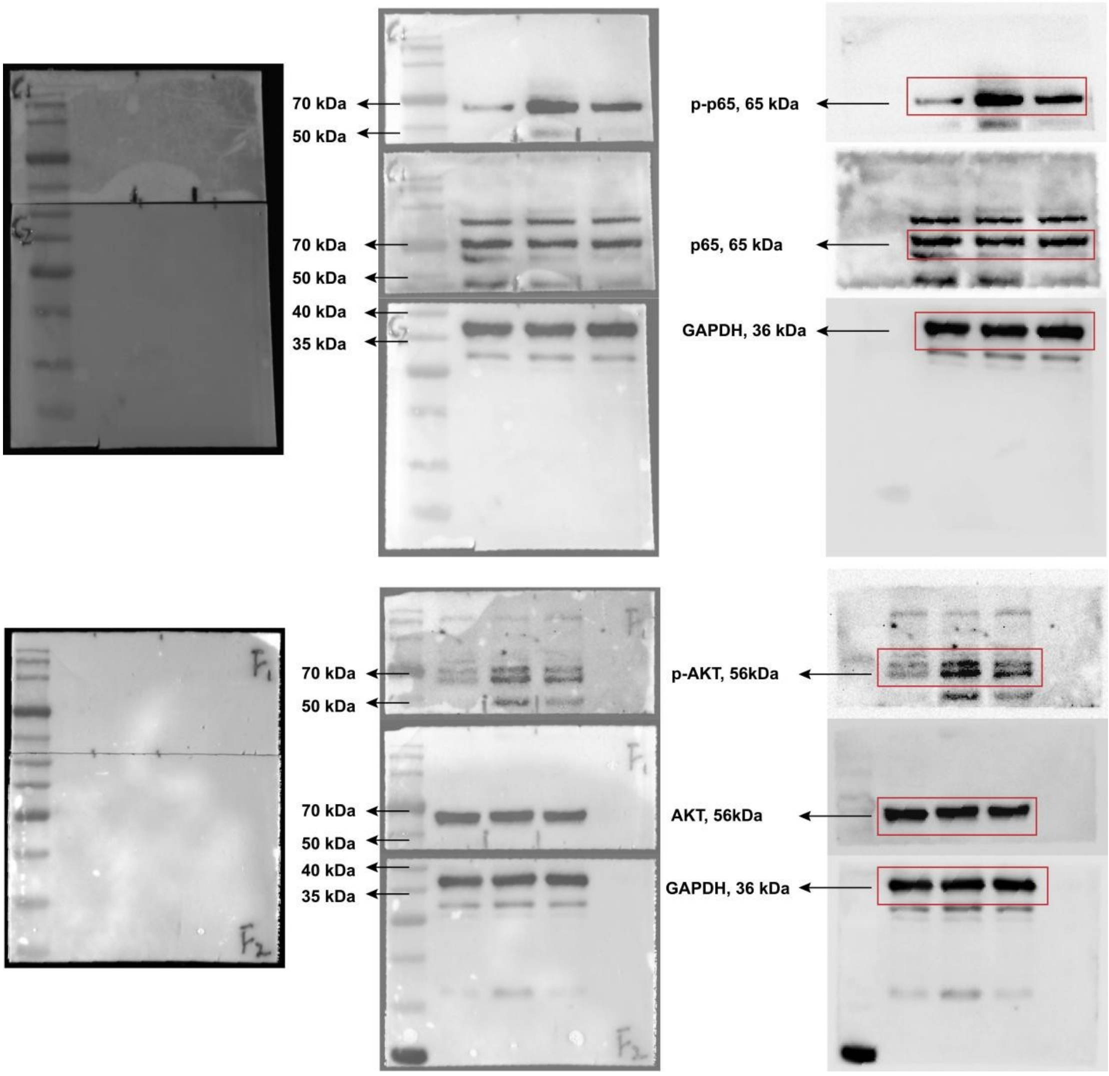

Figure 5E WB data

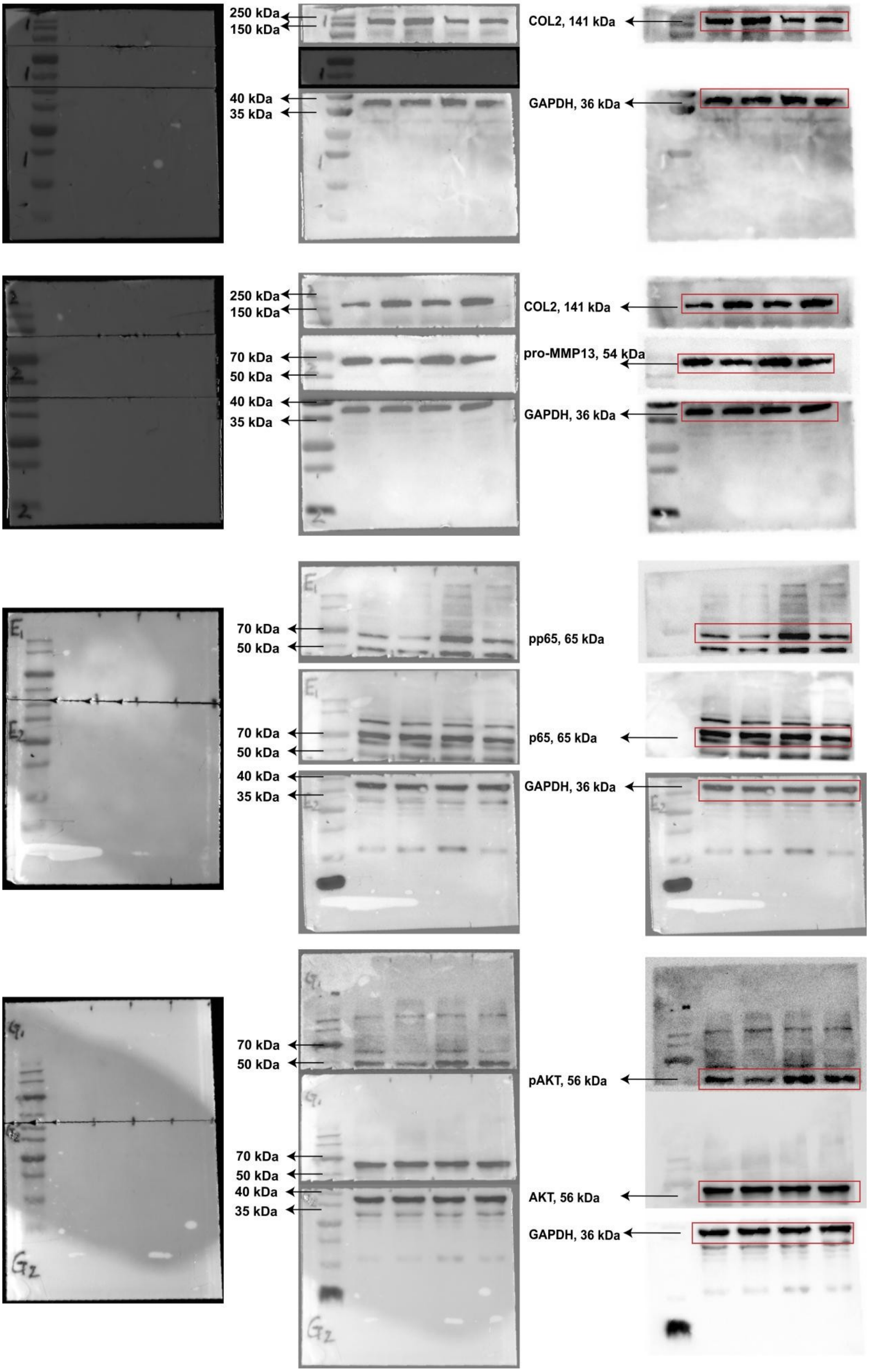

Figure 5K WB data

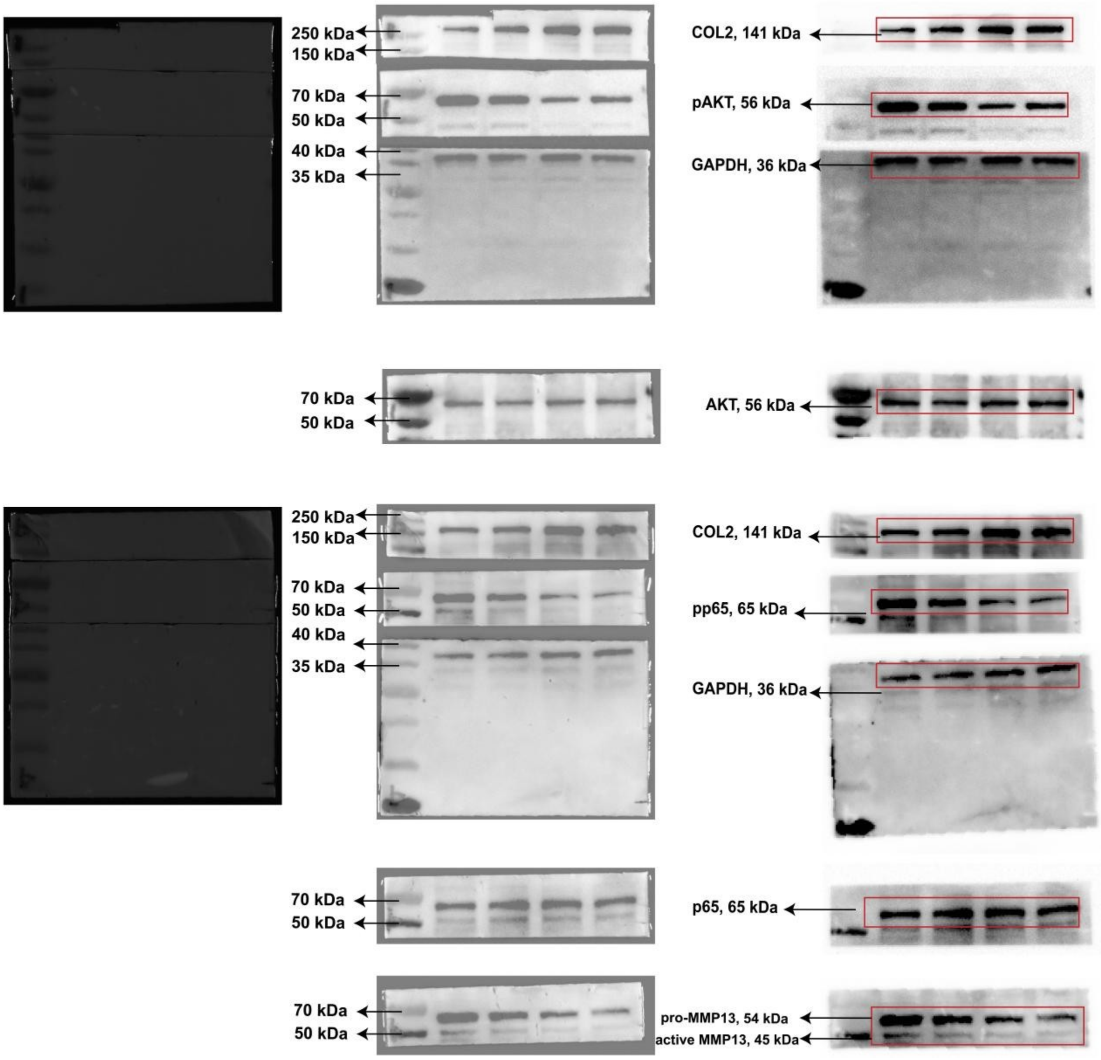

Figure 6G WB data

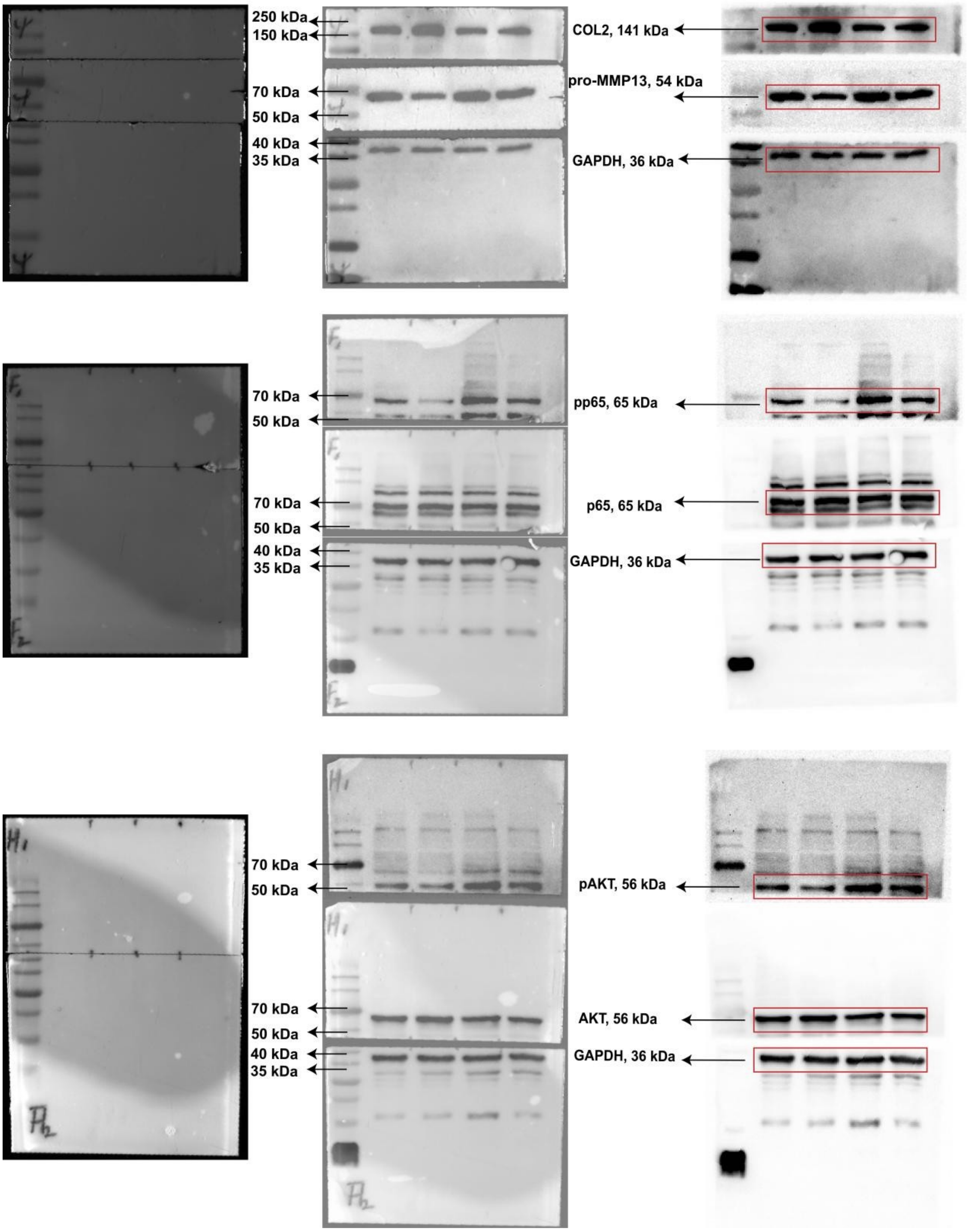

Figure 6H WB data

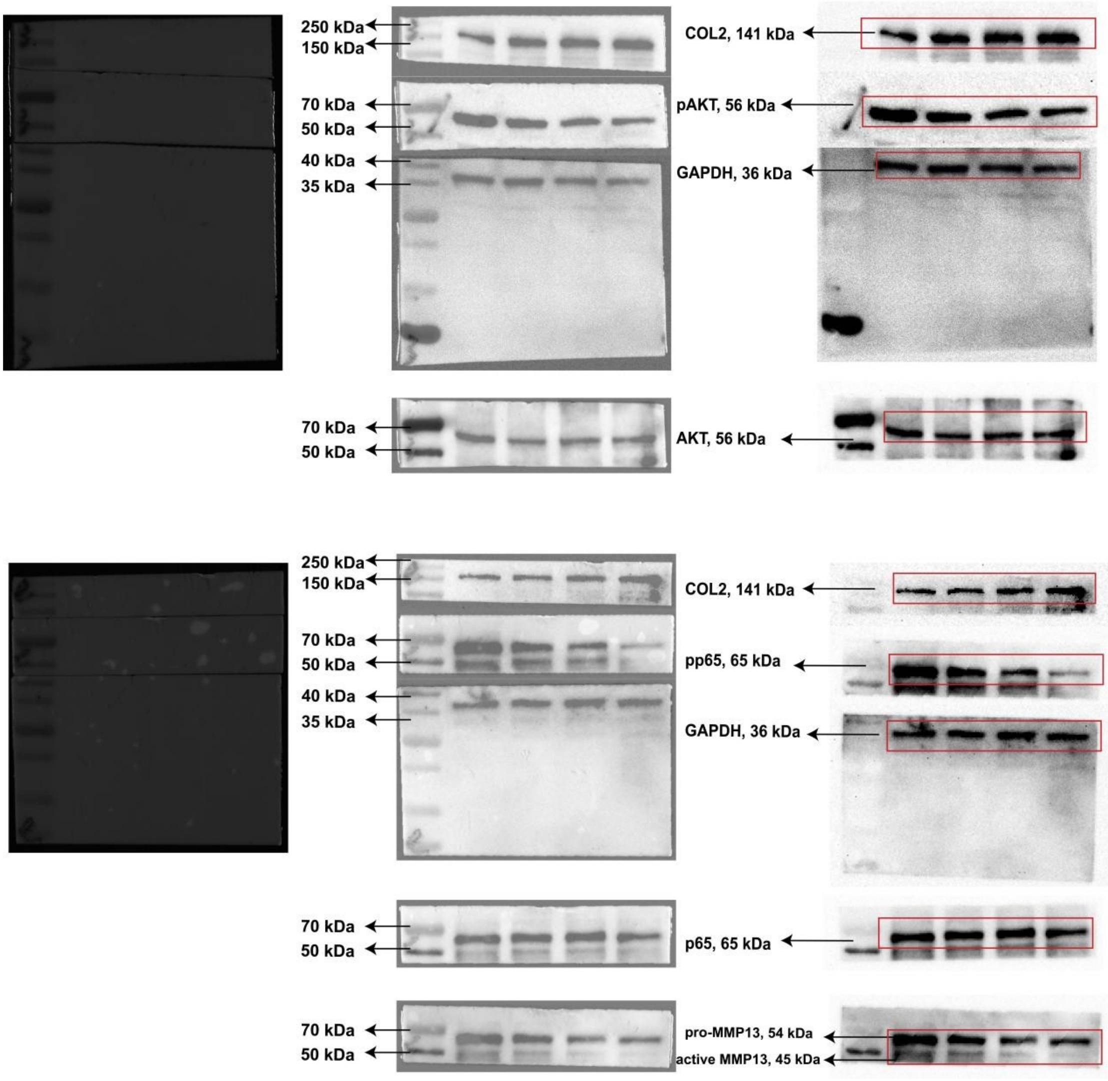

Figure S1 WB data

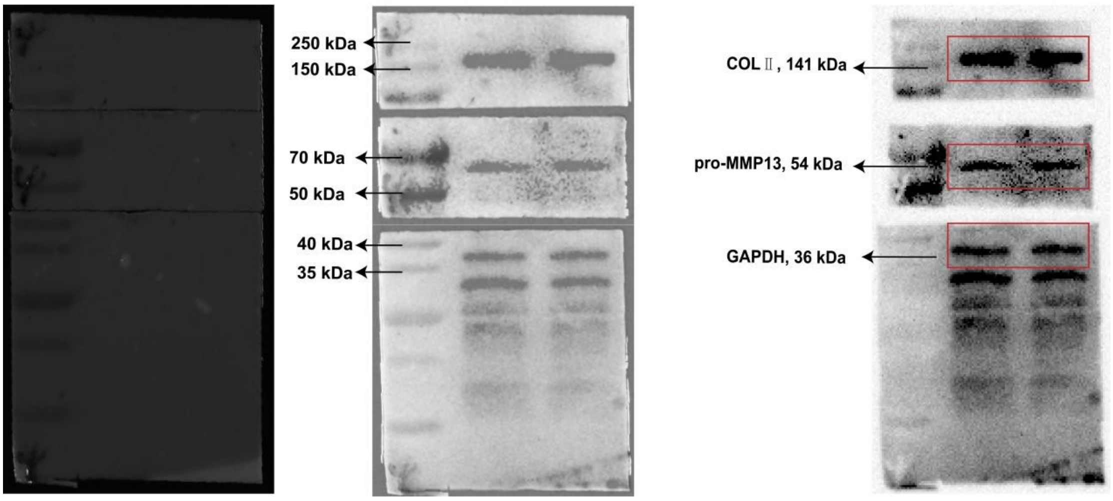

Supplement: Supplementary file 1 [file Table2.docx]
