# Supplementary material for: TEFM drives cartilage degeneration in osteoarthritis via the ROS-AKT/NF-κB signaling axis
Source: Front Immunol. 2026 Oct 7;17:1961836. doi: 10.3389/fimmu.2026.1961836 (PMC13685904; doi:10.3389/fimmu.2026.1961836)
Supplement: Supplementary file 1 [file Table1.docx]

| **Group** | **Sex** | **Age (years)** | **Sampling site** | **Comorbidities** | **KL grade** |
| --- | --- | --- | --- | --- | --- |
| Control1 | Male | 65 | Right fifth finger | None | Grade 0 |
| Control2 | Male | 76 | Right middle finger | None | Grade 0 |
| Control3 | Female | 74 | Left ankle | Hypertension (well-controlled) | Grade 0 |
| Control4 | Male | 63 | Right fifth finger | None | Grade 0 |
| Control5 | Male | 61 | Right ankle | None | Grade 0 |
| Control6 | Male | 68 | Left ankle | None | Grade 0 |
| OA1 | Male | 76 | Right knee | None | Grade 3 |
| OA2 | Female | 65 | Right knee | None | Grade 4 |
| OA3 | Male | 67 | Right knee | None | Grade 3 |
| OA4 | Male | 67 | Left knee | Hypertension (well-controlled) | Grade 3 |
| OA5 | Female | 71 | Left knee | None | Grade 4 |
| OA6 | Male | 64 | Right knee | None | Grade 3 |

**Table S1 Baseline clinical characteristics of human articular cartilage specimens**
